# Supplementary material for: Spatially resolved cell atlas of the teleost telencephalon and deep homology of the vertebrate forebrain
Source: Commun Biol. 2024 May 21;7:612. doi: 10.1038/s42003-024-06315-1 (PMC11109250; doi:10.1038/s42003-024-06315-1)
Supplement: Supplementary file 2 — Supplementary Information [file 42003_2024_6315_MOESM2_ESM.pdf]

Supplementary Text

Supplementary Note 1 - Manual annotation of anatomical regions

Due to the slight angle of the cryosectioning plane, left and the right telencephalic hemispheres captured on each 10μm section differed from each other rostrocaudally. For this reason, each hemisphere was visualized and analyzed separately downstream irrespective of its neighboring hemisphere. Individual hemispheres of coronal sections were organized along the rostrocaudal axis based on tissue cytoarchitectural landmarks using atlases of the *Astotilapia burtoni* cichlid brain as references[1–3]. For each coronal hemisphere, anatomical region identities of spots were manually annotated using Loupe Browser visualization software (10X Genomics). 26 total anatomical regions were annotated based on 1) tissue cytoarchitecture via visual inspection of hematoxylin and eosin (H&E) stained brightfield images of the tissue sections (Fig. S1) and 2) previously established expression of well-studied genes in the subregions of the teleost telencephalon. Annotations of pallial and subpallial subdivisions differ considerably throughout teleost literature, therefore many decisions regarding the boundaries of these regions were based on shared transcriptional patterns revealed by unbiased clustering coupled with cytoarchitectural characteristics observed in H&E images of tissue sections. Within pallial and subpallial areas, further distinction between individual subregions was based on combinatorial gene expression patterns described below and shown in Fig. S6.

In the teleost telencephalon, dorsal/pallial regions and the olfactory bulb glomerular/mitral (or external) layers (OB gml) generally express glutamatergic markers (*slc17a6/vglut.2*, *slc17a7a/vglut.1* (LOC101484681)) while ventral/subpallial regions and the olfactory bulb granule (or internal) cell layer (OB gc) are largely GABAergic (*gad1*, *gad2*)[3]. This trend is reflected in our spatial data. Ventral/subpallial areas (Vv, Vd, Vs, Vc, Vl) and the OB gc expressed *dlx5* and *dlx2*[4]. In the dorsal/pallial regions, we observed expected expression patterns of genes previously described in the teleost pallium, including *eomesa* (LOC101480282)*, neurod1, neurod6b* (LOC101472195)*, emx1* (LOC101483575)*, and bhlhe22,* described below. We note that pallial markers *tbr1b* and *emx3* (LOC101464700) were largely absent from spatial data in contrast with their abundant expression in MC snRNA-seq pallial cell-types[5]. Of the pallial markers, *bhlhe22* was expressed across all dorsal/pallial regions (Dm, Dl, Dc, Dd, Dp)[6]. Spots situated along the periphery of the tissue which expressed strong markers of radial glia cells (RGC) were annotated as belonging to the ventricular zone (VZ), a layer of cells lining the ventricular surfaces (midline and outer brain margins) of the teleost telencephalon. We termed the vVZ as a subset of transcriptionally-distinct spots situated at the ventral ventricular surface.

In the *A. burtoni* cichlid pallium, several major telencephalic divisions, including the Dm, Dc, and Dl, are partitioned into distinct cell groups based on cytoarchitectural properties[1]. The Dc corresponds to several widely-spaced groups of large, migrated cells and the number of Dc subdivisions varies considerably in teleost species. In the *A. burtoni* telencephalon, five Dc subdivisions have been proposed[1], which we used as a guideline for distinguishing general Dc territories in the MC telencephalon, though ours differed slightly based on additional usage of transcriptional profiles to inform decisions. Within the Dm, we identified Dm-1 in the rostral-most coronal section (S_1C_4) and Dm-3 which persisted caudally at the dorsal edge of the pallium. Dm-

2 (Dm-2r, Dm-2c) is proposed as a specialization in cichlids and positioned ventral to Dm-3[1]. Dm-2r corresponded strongly to ST cluster 15 and was identified based on its distinctive shape and ventromedial position below Dm-3/Dc-3 close to the midline, while Dm-2c was only identified in one hemisphere (S_1C_2R) as a tightly packed group of cells lateral to Dm-2r near Dd (Fig. S5). Expression domains of *cnr1* and *epha4b* (LOC101472694) were similar to patterns observed in the zebrafish telencephalon with some exceptions[7]. Expression of *cnr1* was strongest in the Dm-1, Dm-2r, and Dd- a region which is not consistently annotated in teleost studies- while *epha4b* demonstrated higher expression in Dl territories[7]. In line with observations in cichlids[1–3], we identified the Dd as a large, uniform group of cells neighboring the Dm and Dl at rostral levels and positioned dorsal to Dp at caudal levels. The Dl, which demonstrated strong expression of *ucn (uts1)*[8], is a large, laterally positioned region with several distinct cell groups. The Dl-g comprises a group of small, tightly packed cells in the dorsal Dl, and is another putative specialization in the cichlid brain. In other teleost species, Dl divisions are generally divided into dorsal (dDl/Dl-d) and ventral (vDl/Dl-v) subdivisions[1]. Based on H&E images, the Dl-g was identified as largely corresponding to ST cluster 27 and was distinct from Dl-d and Dl-v due to its characteristic tightly packed cells and high expression of *slc17a7a*[3], *pomc* (LOC101475601)[9], and *pvalb7* (LOC101487165)[10,11]. In the area corresponding to the Dl-v, we observed a transcriptionally distinct population of tightly packed cells at its ventral-most pole, which we refer to as the Dl-vv in line with previous annotations in the *A. burtoni* telencephalon[2,12]. Some teleost studies identify a dense cell group in a similar area as the nucleus taenia[1]. The Dp displaced Dl-v at caudal telencephalic levels and strongly expressed *emx1*[3]. The Dl-v/Dl-vv/Dp regions demonstrated strong expression of *eomesa* with lower expression observed in areas corresponding to the Dl-g/Dd. Expression of *neurod6b* was strongest in Dm and Dl-d with lower expression in the Dd and Dp[6], while *neurod1* was expressed in the Dl-v, Dl-vv and Dp[4].

Several divisions in the ventral telencephalon were clearly distinguished due to well-studied gene expression patterns and distinct cytoarchitectural features. The ventrally-located Vv was identified based on expression of several genes, including *isl1a* (LOC101475168)[4] and *nkx2.1*[4]. At caudal levels in the teleost telencephalon, a preoptic region is located in the ventral-most position along the midline, but due to the angling of the sectioning plane this region was not included in our sections. Dorsal to Vv, the Vd was divided into a rostral part (Vd-r) and caudal part (Vd-c) in line with observations in the *A. burtoni* telencephalon[1]. The Vs is positioned dorsal to the Vd and ventral to Dm along the midline and divided into lateral and medial parts, while a division referred to as the Vp appears below the Vs at caudal levels[1]. Instead, we annotated the MC Vs as one unit and did not distinguish a separate Vp. We examined expression of several genes that mark mammalian principal striatal neurons (medium spiny neurons, MSNs) that have been studied in teleosts, including *penka* (LOC101474395), *tac1*, *six3a*, and *sp9*. The Vd and Vs demonstrated high expression of *six3a*[6], and the border between these regions was partially determined based on the distribution of *penka,* expressed strongly in the Vd*,* and *tac1*, which is more abundant in the Vs[7,13]. Strong expression of *sp9* was observed in the Vd and Vc[6], which is a small, migrated group of cells lateral to Vv and Vd. This region was easily identified in the H&E images of many sections and corresponded largely with ST cluster 6 (Fig. S5). In the cichlid subpallium, two other migrated groups of cells have been identified- the Vl and Vi[1]. We

observed strong co-localization of *sst1.1* (LOC101485677) and *npy* in the Vl[1415], the most lateral division of the ventral telencephalon, which additionally exhibited high expression of *crhb*[8]. The Vi was identified in one caudal hemisphere (S_2C_3R) as a small cluster of cells located centrally in a largely cell-poor region of the subpallium that we refer to as the *Vx*. Additionally, we compared expression of dopaminergic genes (*th*, *slc6a3 (dat)*, *slc18a2 (vmat)*) in ST data with patterns described in teleosts to further classify the Vd, Vc, and OB gc[1617] and observed expected expression of dopamine receptor *drd2a* in the Vd, Vc, Vv, Vi, OB, and Dp[17].

Further determination of regional identities was based on expression patterns of transcriptional regulators. We examined the distribution of Zic family genes and observed strong expression of *zic1* in the Vv, expression of *zic3* in the Dl-v, Dp, Vv and Vc, and expression of *zic2* in the Vv, Dp, and Dl-v[6]. LIM homeobox genes *lhx6a* and *lhx8 (lhx7)* were expressed strongly in the Vv[18], while *lhx9* was localized in the OB gml[19] and Dp. Inhibitor of DNA Binding (ID) family members demonstrated expression patterns in line with observations in the adult teleost telencephalon, including *id1*, which is expressed in radial glia cells (RGCs)[20] and was localized along the VZ. The Dm, Dc, and Dp regions demonstrated strong expression of *id2a* while *id4* (LOC101483990) was expressed most strongly in the Vd and Vv, with lower expression in Dm-3 and other subpallial regions[20]. Furthermore, we observed localized expression of *vax1* in the Vc and *prdm12b* and *prdm16* in the Vv, genes which are exclusively expressed in these locations in the zebrafish telencephalon[6].

Supplementary Note 2 - Comparison of manual and computational cell abundance estimation in spatial transcriptomics spots

To determine the rough accuracy of computational cell abundance estimation performed by cell2location[21], cells were manually counted in a select tissue hemisphere (S_1C_2R) and compared (Fig. S7). Generally, both methods identify a large density of cells near the peripheral edges of the tissue and fewer cells in the middle. The Pearson and Spearman correlation coefficients were calculated to quantify this relationship. There was moderate-high agreement as the Pearson correlation was 0.3989124 (Pearson's correlation coefficient p-value=1.407487e-09) and the Spearman correlation was 0.4973912 (Spearman's correlation coefficient p-value=8.943762e-15; Fig. S7b).

Supplementary Note 3 - Correlations of expression of common marker genes in cichlid and mouse cell-types

Several consistent cell-type relationships were observed using both the SAMap approach and correlation approach (Fig. S9), including the following. 4_GABA (*six3a*+) cichlid cell-types in the Vd and Vs were strongly correlated with mouse striatal MSNs (MSN1-4). Cichlid 5_GABA inhibitory populations located in the OB were strongly correlated with their mammalian counterparts (OBINH1-4). Cichlid 6_GABA and 15.3_GABA were significantly correlated with MGE interneurons (TEINH17-19 and TEINH21).

Cichlid excitatory neuronal populations located in the Dl-g (8.1_Glut, 8.2_Glut and 8.8_Glut) had the greatest and significant similarity to mouse cells from pyramidal layer 6 of the cortex. Additionally, another Dl-g cell-type (12_Glut) was similar to several neocortical populations

(TEGLU7-9). Most cichlid Dl-v cell-types (8.3_Glut, 8.5_Glut, and 8.9_Glut) have significant correlations with excitatory neurons in the hippocampus (TEGLU23-24). Cichlid 11.1_Glut located in the Dp was significantly correlated with piriform pyramidal cells (TEGLU17).

Supplementary Note 4 - Analysis of common marker genes and SAMap driving genes in cichlid-to-mouse cell-type comparisons

To robustly determine molecular signatures of conserved cell-types found by SAMap, we used two methods: conserved marker genes (differentially expressed genes (DEGs) in common between cell-types) and genes found by SAMap that drive relationships. Both methods require that each gene is differentially expressed, however the thresholds used are different. The former is more strict (see Methods) and is orthogonal to SAMap. Cichlid-mouse cell-type pairs exhibiting significant similarity had a mean of 78.3±2.54 shared marker genes and a mean of 451±24.0 SAMap driving genes (Supplementary Dataset 7), both of which were significantly greater than in non-significant cell-type pairs (Welch Two Sample t-test; $p_{markers}$=3.79e-49, $t_{markers}$=19.6; p_{SAMap}=2.13e-30, t_{SAMap}=13.8; Fig. 4d-e). Cell-type specific expression of genes driving select significant cell-type pairs is shown in Figure 4f.

Supplementary Note 5 - SAMap comparison of cichlid cell-types to an additional mouse telencephalon scRNA-seq dataset

Another scRNA-seq dataset from the mouse telencephalon[22] (Saunders et al. 2018) was used to determine the robustness of observed similarities between cichlid and mouse cell-types (Fig. S11). In this comparison, the mouse dataset consisted of regions annotated as Frontal Cortex (FC), Globus Pallidus (GP), Hippocampus (HC), Posterior Cortex (PC) and Striatum (STR).

Cichlid 1.1_RG expressed many markers of neurogenesis such as *sox4* and demonstrated transcriptional similarities to mouse cell-types with a class label of "Neurogenesis" given by Saunders et al. Some examples of mammalian neurogenic cell-types similar to cichlid 1.1_RG included: HC_13-1 (SGZ), HC_13-2 (SGZ), HC_13-3 (SGZ), HC_13-4 (SGZ), HC_13-6 (SGZ), STR_2-1 (RMS), STR_2-2 (RMS), STR_2-3 (RMS), STR_2-4 (RMS), STR_2-5 (RMS), STR_2-6 (RMS) and mitotic GP_4-6. As a *fabp7*+ radial glia cell-type, 1.1_RG showed transcriptional similarity to the *Fabp7*+ astrocyte cell-type HC_7-3. Cichlid 1.1_RG and cichlid 1.2_RG showed transcriptional similarity to the following astrocyte cell-types: FC_8-1, FC_8-2, GP_5-1, GP_5-2, HC_7-1, HC_7-2, PC_8-1, PC_8-2 and PC_8-3. However 1.2_RG generally showed greater similarity to these astrocyte cell-types except for *Fabp7*+ FC_8-2.

Cichlid *tnr*+ 2.1_OPC was transcriptionally similar to *Tnr*+ polydendrocyte cell-types: PC_10-1, PC_10-3, PC_10-4, GP_4-1, GP_4-4, GP_4-5, GP_4-7, FC_10-1, FC_10-3, FC_10-5, FC_10-6, HC_9-1, HC_9-2, HC_9-3, HC_9-4, STR_5-1, STR_5-2 and STR_5-3. Cichlid 2.2_Oligo was transcriptionally similar to oligodendrocyte cell-types: PC_9-2, PC_9-3, PC_9-4, PC_9-5, FC_9-1, FC_9-3, FC_9-4, FC_9-5, GP_10-1, GP_10-2, GP_10-3, GP_10-4, GP_10-5, GP_10-6, HC_8-1, HC_8-2, HC_8-3, HC_8-4, STR_3-1, STR_3-2 and STR_3-3. Cichlid 1.3_MG was transcriptionally similar to microglia/macrophages: PC_11-1, PC_11-2, PC_11-3, PC_11-4, FC_11-1, FC_11-3, FC_11-4, GP_11-1, GP_11-2, HC_10-1, HC_10-2, STR_6-1 and STR_6-2. Cichlid 3_Peri was transcriptionally similar to cells from the endothelial tip: PC_14-3, PC_14-4,

PC_14-5, FC_14-3, FC_14-4, FC_14-5, GP_9-1, GP_9-2, GP_9-3, HC_17-1, HC_17-2, HC_17-3 and HC_17-4.

Cichlid *sox4*+ 9.5_Glut was transcriptionally similar to neurogenic *Sox4*+ neurons from the hippocampus: HC_13-5 (SGZ) and HC_13-6 (SGZ). Cichlid 15.7_Glut was the reciprocal top hit of Cajal-Retzius cells in the frontal cortex.

This mouse dataset contained few *Th*+ dopaminergic inhibitory interneurons (STR_13-5 and STR_14-4), but cichlid 5_GABA cell-types were significantly similar to them, as well as to other striatal inhibitory interneurons. 6_GABA was transcriptionally similar to many *Pvalb*+ inhibitory interneurons: PC_5-1, PC_5-2, PC_5-3, FC_2-6, FC_2-7, FC_2-9, FC_2-10, HC_1-6 and HC_1-7. Additionally, 15.3_GABA was transcriptionally similar to MGE-derived cell-types that were *Npy*+ (STR_15-1) and *Sst*+ (PC_5-6, FC_2-1, FC_2-2, FC_2-3, FC_2-4, HC_1-3, HC_1-4, HC_1-5, HC_1-9, STR_15-1). 15.1_GABA/Glut, 15.2_GABA, and 15.4_GABA were transcriptionally similar to an *Lhx6*-enriched cell-type in the globus pallidus externus, as well as to many other globus pallidus cell-types. Cichlid inhibitory 4.1_GABA had significant transcriptional similarity to all of the following striatal spiny neurons cell-types, and several other cichlid cell-types had significant similarities to some of the following striatal spiny neurons cell-types: GP_3-1, GP_3-2, GP_3-3, GP_3-4, GP_3-5, GP_3-6, STR_10-1, STR_10-2, STR_10-3, STR_10-4, STR_10-5, STR_11-1, STR_11-2, and STR_11-3.

Cichlid excitatory neuronal populations located in the Dl-g (8.1_Glut, 8.2_Glut, 8.8_Glut, and 12_Glut) had the greatest and significant similarity to retrosplenial areas (PC_6-1, PC_6-2, PC_6-5, and PC_6-6), entorhinal cortex (PC_6-7 and HC_5-11) and other neocortical cell-types (PC_2-1, PC_2-2, PC_2-4, PC_2-5, PC_2-6, PC_2-7, PC_2-10, PC_2-11, PC_2-16 and PC_2-18). Cichlid 11.1_Glut located in the Dp had the greatest similarity to deep layer pyramidal cells (FC_6-6). Cichlid Dl-v cell-types (including 8.3_Glut and 8.5_Glut) had significant similarity to lateral CA3 principal cells (HC_6-3), but 8.3_Glut was the strongest. Additionally, 8.3_Glut showed strong similarity to many other hippocampal populations (HC_5-4, HC_5-5, HC_5-6, HC_5-9, HC_6-1, HC_6-2, HC_6-3, HC_6-4, HC_6-5, HC_6-6, HC_6-7 and HC_6-8).

<u>Supplementary Note 6 - SAMap comparison of cichlid and goldfish cell-types</u>
Teleosts are a diverse clade and have a long evolutionary history, here we compare our atlas to that of another teleost species (Fig. S12). Recently, the cell-types in the goldfish telencephalon have been profiled[23] and compared to cell-types in the mouse forebrain[24]. This goldfish atlas contains 83,651 genes (NCBI Assembly ASM336829v1), perhaps due to genome duplication, and the atlas contains 106 cell-types, ensuring many-to-one mapping with cichlid (53 cell types). Furthermore, in our comparison of the cichlid telencephalon to the mouse forebrain, only cell-types from the mouse telencephalon were included, while in the comparison of the goldfish telencephalon, additional mouse brain regions were used, including the diencephalon. There were differences in the approach taken in predicting the anatomical location of cell-types profiled by scRNA-seq in spatial transcriptomics data. The location of cichlid cell-types were predicted by cell2location, while the location of goldfish cell-types were determined by a custom mapping score. Despite these differences in approach, the evolutionary distance between these

species and differences in the manual anatomical annotations between cichlid and goldfish spatial atlases, we find many conserved cell-types within these teleost species, as well as conserved cell-types between both teleosts species and mice.

Our analysis revealed a strong correspondence between non-neuronal populations in cichlids and goldfish, including glial-like cells (cichlid 1.1_RG and goldfish Ependymoglia-HES5-DLL1; cichlid 1.2_RG and goldfish Ependymoglia-MFGE8-MT4), oligodendrocytes (cichlid 2.1_OPC and goldfish OPC; cichlid 2.2_Olig and goldfish Oligodendrocytes), macrophages (cichlid 1.3_MG and goldfish Macrophages-MAFB-MRC2), endothelial cells (cichlid 3_Peri and goldfish EC). Consistent with the strong correspondence of non-neuronal populations within teleosts, these cell-types also showed strong correspondence to the mammalian populations in their respective pairwise comparisons (Fig. 4 and Tibi et al. 2023[23]). In both comparisons *sox4*+/*mex3a*+ teleost neuroblast populations, which mapped to each other, showed strong similarities to neurogenic populations from the mammalian dentate gyrus (cichlid 9.5_Glut with mouse DGNBL1 and goldfish GLUT-26-MEX3A-TUBB5 with mouse CR).

GABAergic neurons demonstrated strong correspondence between teleost species. Cichlid 4_GABA cell-types showed significant similarity to goldfish GABAergic cell-types that mapped to mouse MSNs (goldfish GABA-24-30). This pattern was most consistent with cichlid 4.1 and 4.2_GABA, which also showed consistent similarity to LGE-like populations in other vertebrates (Fig. 5). Additionally, a subclass of MSN neurons appear to be conserved across teleost lineages and mammals. Cichlid 7_GABA was significantly similar to goldfish GABA-23-MEX3A-TUBB5,and both teleost cell-types mapped to mammalian MSN5. Furthermore, LGE-derived *th*+ olfactory inhibitory interneuron-like populations appears strongly conserved across cichlid, goldfish, and mouse (cichlid 5.1 and 5.2_GABA, goldfish GABA-15-TH-SLC6A3 and mouse OBDOP1 and OBDOP2).

Consistent similarities were observed between other GABAergic classes in teleosts and mammals, including *sst*+ MGE-like interneurons. In both teleost species, *sst*+ cell-types were significantly similar and mapped strongly to mammalian MGE-derived populations (cichlid 15.3_GABA, goldfish GABA-8-10, and mouse TEINH19 and TEINH21). Based on spatial transcriptomics data, these cell-types were located in similar regions of the teleost subpallium (labeled as Vl and Vx in cichlids and Vsst in goldfish). However, differences were observed in populations resembling mammalian septal nuclei. Putative Vv-located cichlid 15_GABA/Glut cell-types showed strongest similarity to goldfish cell-types that mainly mapped to the mammalian cell-types located in the diencephalon (DEINH2-8). Mouse cell-types that were annotated as derived from the diencephalon were excluded from our cross-species comparison between cichlid and mouse telencephalic cell-types.

While we observed strong correspondence between cichlid and goldfish glutamatergic cell-types, we note that some populations did not consistently map to the same cell-types when compared to the mouse dataset. Cichlid Dl-g cell-types 8.1_Glut and 8.2_Glut were transcriptionally similar to mouse cell-types from cingulate/retrosplenial areas and goldfish GLUT-11-SHC1-CCK, while independent analysis reported that the latter mapped to neurons

from the mouse diencephalon, which was not included in our comparison. However, several cell-types from both teleost species with significant similarity to one another (cichlid 8.7_Glut and 8.10_Glut with goldfish GLUT-2-CBLN1-EMX1 and GLUT-3-CBLN1-OPCML) consistently mapped to the cell-types from mouse subiculum/entorhinal regions (TEGLU5 and TEGLU21 respectively). Similarly, teleost cell-types that were transcriptionally similar to mouse CA3 cell-type, TEGLU23, showed strong similarity to one another, including cichlid 8.6 and 8.9_Glut with goldfish GLUT-42-POU3F3-POSTN. Lastly, cichlid cell-type 13_Glut putatively located in the Dm-2r region was significantly similar to goldfish GLUT-8-PAPPA2-NR2F2. The cichlid Dm-2r demonstrated transcriptional similarity to pallial amygdala-like cell-types and regions in turtles, and the goldfish cell-type GLUT-8-PAPPA2-NR2F2 mapped to an amygdala cell-type in mammals.

<u>Supplementary Note 7 - Analysis of common marker genes and SAMap driving genes in cichlid-to-mouse brain region comparisons</u>

Again, we found that brain region pairs demonstrating significant transcriptional similarity exhibited significantly more shared marker genes (Welch Two Sample t-test; p_{turtle}=2.39e-07, t_{turtle}=5.68; p_{mouse}=4.94e-04, t_{mouse}=4.03) and SAMap driving genes than other brain region pairs (Welch Two Sample t-test; p_{turtle}=4.87e-06, t_{turtle}=4.89; mouse: p_{mouse}=0.00375, t_{mouse}=3.32; Fig. S17; Supplementary Dataset S12). The molecular similarity of these regions was supported by the sum of the normalized expression of their shared marker genes (Fig. 6c-d).

<u>Supplementary Note 8 - Genes driving effects between anatomical regions in the cichlid and mouse telencephalon</u>

Cichlid OB regions (OB gc, OB gml) demonstrated strong transcriptional similarities with the mouse main olfactory bulb (MOB) and genes that contributed to this pairing included *gpsm1*, identified as a novel marker of olfactory bulb granule cells in the mouse[25], and *arx*, which is critical for OB development and differentiation of *Th*+ neurons in this region in the mouse brain[26]. Other genes included markers of OB dopaminergic cells (*th, sall3*)[27] and genes involved in OB development and function (*dlx2, meis2, sp8b, zic1, zic3*)[28].

Multiple cichlid regions mapped to the mouse nucleus accumbens (ACB) of the ventral striatum, including the Vd-c, Vd-r, and Vs. The vast majority of cells in the mammalian nucleus accumbens are medium spiny neurons (MSNs), and many genes that contributed to cichlid region-mouse ACB pairings are expressed in MSNs, including *bcl11b*, *baiap3*, *cpne5*, *id4*, *slc2a13*, *slc32a1*, *flrt3*, *hs6st2*, and *pbx3*[29]. Furthermore, genes enriched in specific MSN subtypes (D1 MSN-enriched *tac1* and D2 MSN-enriched *sp9*)[29] were also included in ACB pairings.

The cichlid Vv demonstrated strongest transcriptional similarity to the mouse lateral septal complex (LSX), as did the Vc. Several genes driving the Vv-LSX pairing are expressed in the adult murine lateral septum, including *nts*[30], *pnocb*[31], *nrxn3*[32] and *zic5*[33], while others have been shown to play important roles in the development of septal populations, including *isl1a*[34], *prdm12b*[35], *zic1*[36,37] and *zic3*[37]. The Vv additionally mapped to the mouse pallidum (PAL) along with the Vl. According to the Allen Mouse Brain Reference Atlas[38], the PAL contains regions

such as the globus pallidus, medial septal complex, and the bed nuclei of the stria terminalis, among others. Several genes that contributed to the Vv-PAL pairing included *pvalb6* and *zic1*, which are expressed in the adult mouse medial septum[36], and *lhx8*, which plays a role in development of medial septum cholinergic neurons which project to the hippocampus[39].

The mouse hippocampal CA3 region was transcriptionally similar to Dl-vv and two pallial regions in the cichlid rostral-most sections, the Dm-1 (S_1C_4) and Dl-d (S_1C_4L). Many genes driving the Dl-vv to CA3 pairing have been identified as enriched in the mouse hippocampal CA3 based on DNA microarray data (*grm1*, *bdnf*)[40] and single cell RNA-seq (*grm1*, *bdnf*, *rasgrp1*, *ptk2b*, *prkce*, *pclo*, *cpe*, *spock1*, and *prkca*)[41].

The cichlid Dl-g mapped to the visual areas of the mouse isocortex (VIS). A subset of genes driving this comparison are reported as highly expressed in the mouse cerebral cortex (*camk2g*, *dusp6*, *egr1*, *gabra1*, *gadd45b*, *kcnf1*, *ncald*, *nrn1*)[42]. Furthermore, several genes driving the Dl-g to VIS pairing demonstrate layer-specific enriched expression in a transcriptomic atlas of mouse cortical layers (L2/3: *adgrb1*, *atp1a1*, *fosl2*, *nrn1*; L2/3 and L4: *golga7b*, *nr2f1*, *r3hdm1*; L5: *bhlhe40*, *camk2g*, *dusp6*, *gabra1*, *gfra2*; L6: *ncald*)[43].

Supplementary Note 9 - Representation of different gene classes among driving genes for cichlid, turtle, and mouse cortical cell-types and regions

The composition of gene classes (transcription factors, neuromodulatory ligands, neuromodulatory receptors) driving significant cell-type relationships did not differ between the aDC with the neocortex, aDVR with the neocortex, and other significant cell-type pairs (Fig. 7b; χ^2 test, p_{tf}=0.290, t_{tf}=8.50; p_{ligand}=0.839, t_{ligand}=3.47; $p_{receptor}$=0.806, $t_{receptor}$=3.77; Supplementary Dataset 13). Similarly, genes driving effects between the cichlid Dl-g and mammalian neocortex cell-types did not differ in composition compared to other significant cichlid-mouse cell-type pairs (Fig. 7c; χ2 test, p_{tf}=0.481, t_{tf}=0.498; p_{ligand}=0.757, t_{ligand}=0.0958; $p_{receptor}$=0.1721075, $t_{receptor}$=1.86). Additionally, genes driving effects between the cichlid Dl-g and turtle aDC, cichlid Dl-g and turtle aDVR, and other significant cell-type pairs differed only in composition of neuromodulatory ligands (Fig. 7d, χ2 test, p_{tf}=0.634, t_{tf}=2.56; p_{ligand}=0.0381, t_{ligand}=10.1; $p_{receptor}$=0.233, $t_{receptor}$=5.58). In summary, transcriptional similarities between cell types in these brain regions found by SAMap do not exhibit bias in gene class composition.

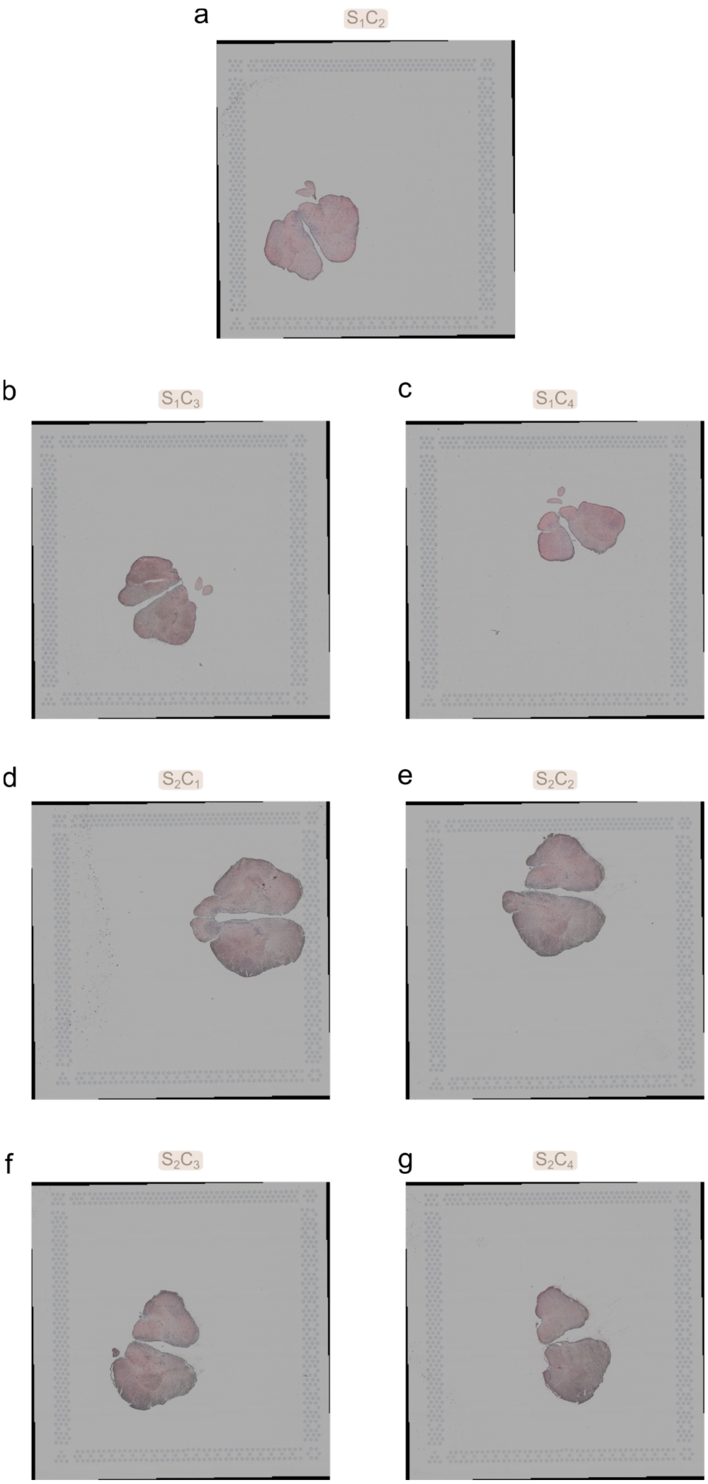

Figure S1. Images of sequenced coronal sections of the cichlid telencephalon. a-g) Brightfield images of H&E-stained slides for each capture area from each subject (S_1C_{2-4} and S_2C_{1-4}), respectively.

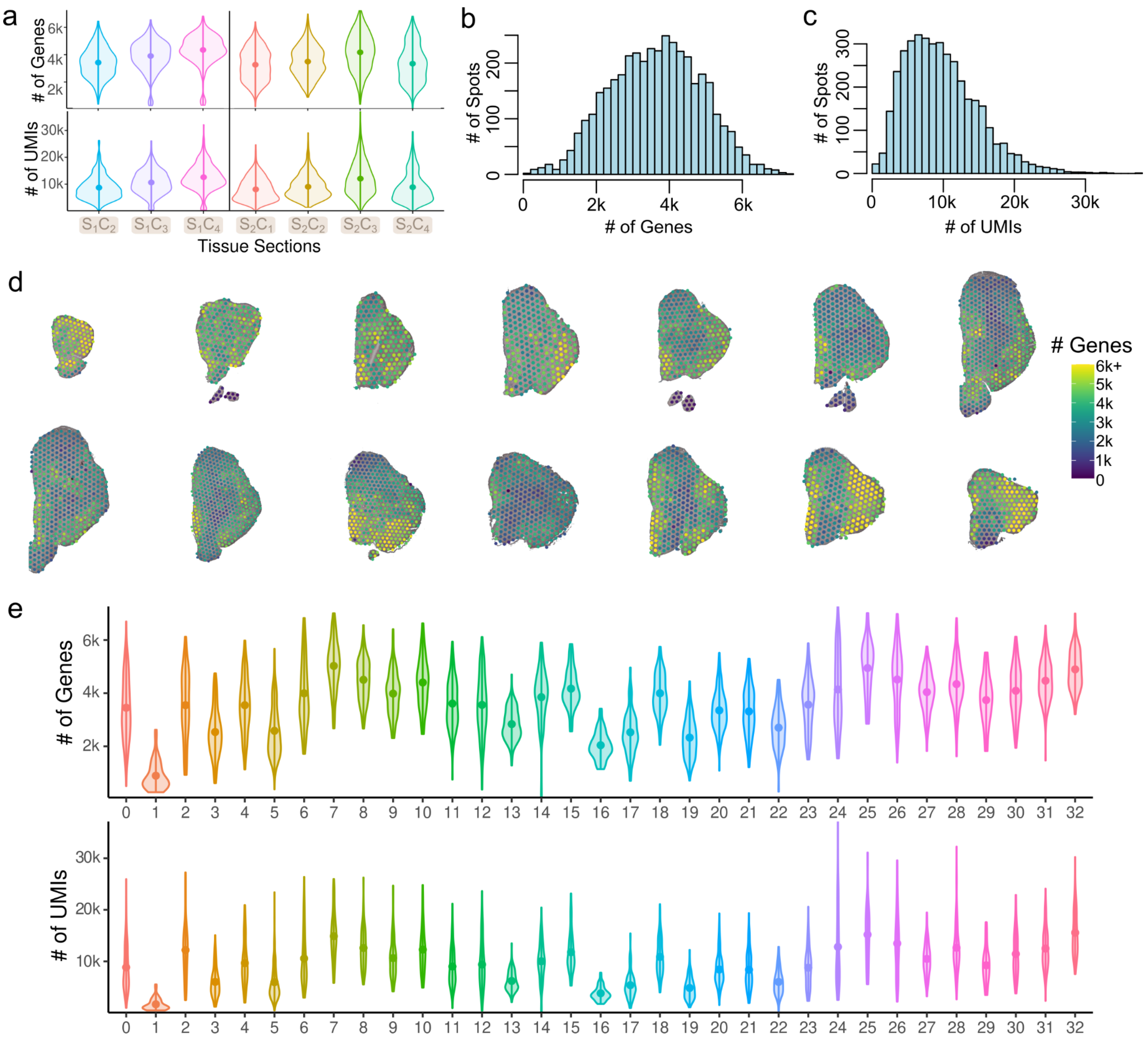

Figure S2. Spatial transcriptomics data quality. a) Distribution of the number of genes and UMIs across capture areas from subjects represented by a violin plot. Dots signify the mean value per capture area and vertical lines represent the range. **b-c)** Distribution of number of genes and UMIs across all subjects, respectively. **d)** The number of genes expressed in each spot shown on the tissue. **e)** Distribution of the number of genes and UMIs across clusters represented by a violin plot. Dots signify the mean value per cluster and vertical lines represent the range.

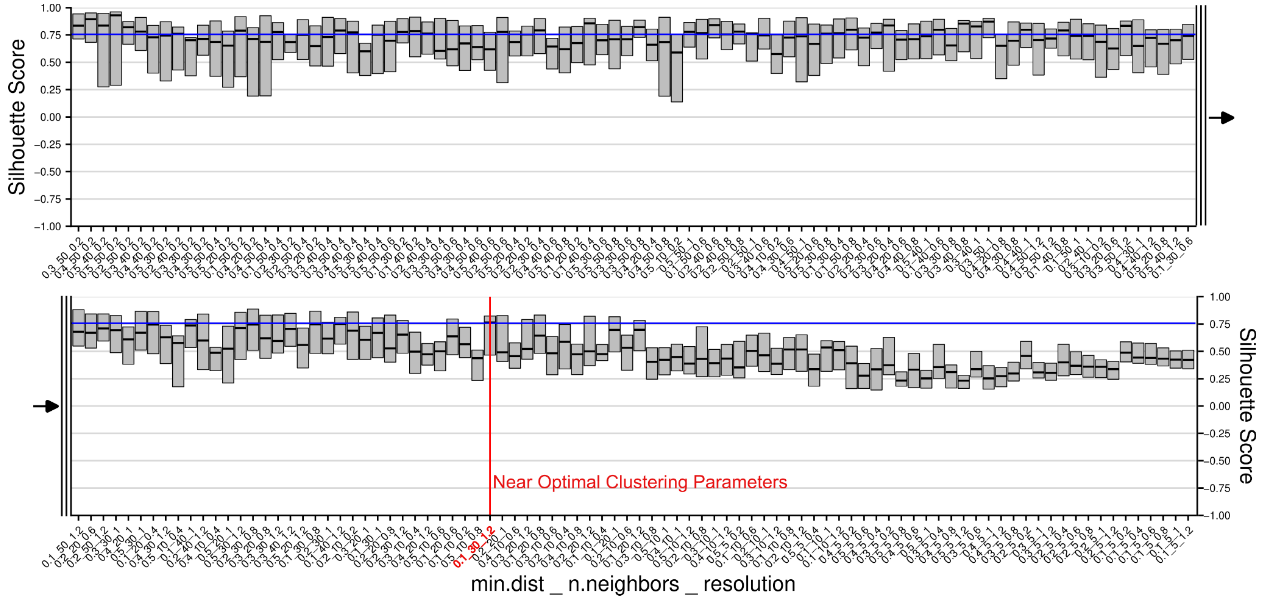

Figure S3. Near-optimal clustering parameters of spatial transcriptomics data. Silhouette scores shown on the y-axis for combinations of clustering parameters shown on the x-axis. Boxplots represent the silhouette scores of bootstraps of the data with medians shown as lines and shaded regions as the 95% confidence interval. ChooseR determines the near-optimal combination (red line) as the one yielding the highest number of clusters whose median silhouette score is greater than the highest lower bound of the 95% confidence interval (blue line). Combinations of values were evaluated for min.dist, n.neighbors and resolution (see Methods).

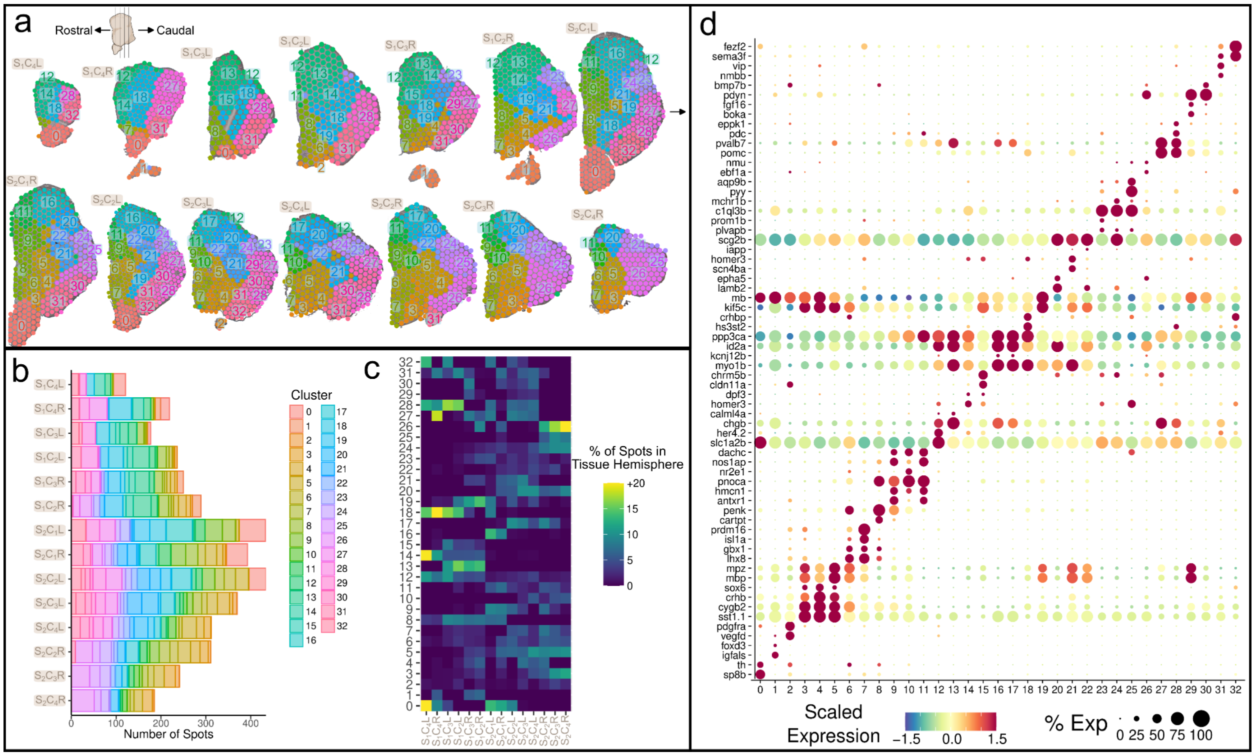

Figure S4. Unbiased clusters of spatial transcriptomics data. a) Spots on tissue colored by clusters with tissue hemispheres ordered rostrocaudally. **b)** Number of spots from each cluster in tissue hemispheres. **c)** Composition of spots from tissue hemispheres by clusters. **d)** Select top marker genes of clusters. Dots are colored by their mean scaled expression in each cluster and dot size indicates the percent of cells in clusters expressing the marker.

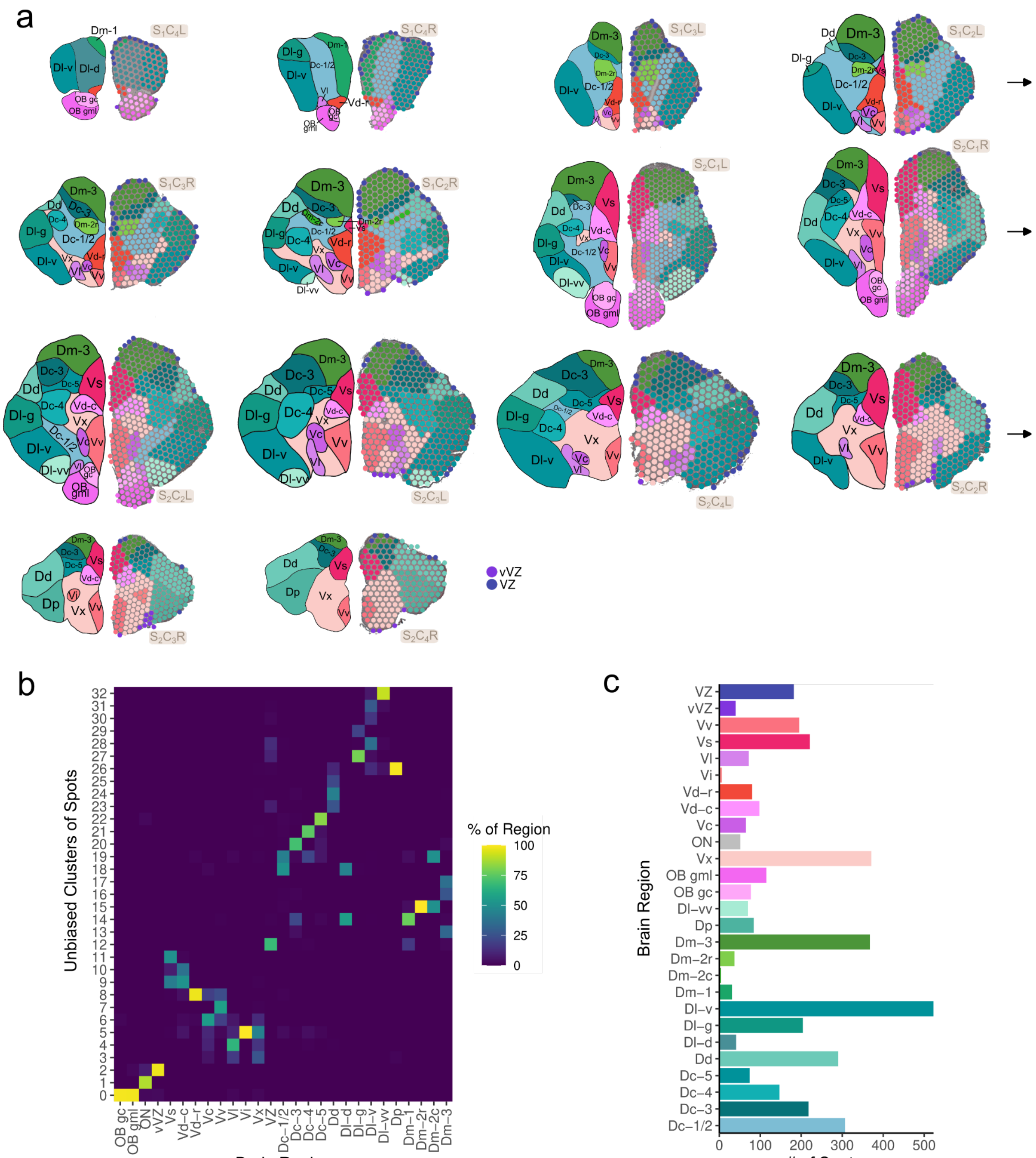

Figure S5. Manually annotated anatomical labels of spots. a) Annotations of anatomical subregions of the teleost telencephalon on tissue images. Each tissue hemisphere is represented on the left by an illustration of the tissue with anatomical subregions labeled and on the right by the tissue images with spots colored by anatomical labels. Dc=central division of D, Dc-1,2,3,4,5=subdivisions of Dc, Dd=dorsal division of D, Dl=lateral division of D, Dl-d=dorsal subdivision of Dl, Dl-g=granular zone of Dl, Dl-v=ventral subdivision of Dl, Dl-vv=ventral zone of Dl-v, Dm=medial division of D, Dm-1,2,3=subdivisions of Dm, Dm-2c=caudal part of Dm-2, Dm-

2r=rostral part of Dm-2, Dp=posterior division of D, OB gc=olfactory bulb granule (internal) cell layer, OB gml=olfactory bulb glomerular and mitral (external) cell layers, Vc=central nucleus of V, Vd=dorsal nucleus of V, Vd-c=caudal part of Vd, Vd-r=rostral part of Vd, Vi=intermediate nucleus of V, Vl=lateral nucleus of V, Vs=supracommissural nucleus of V, Vv=ventral nucleus of V, Vx=unassigned subdivision of V, VZ=ventricular zone, vVZ=ventral ventricular zone, ON=olfactory nerve. **b)** Composition of anatomical subregions by unbiased clusters. **c)** Number of spots per anatomical subregion.

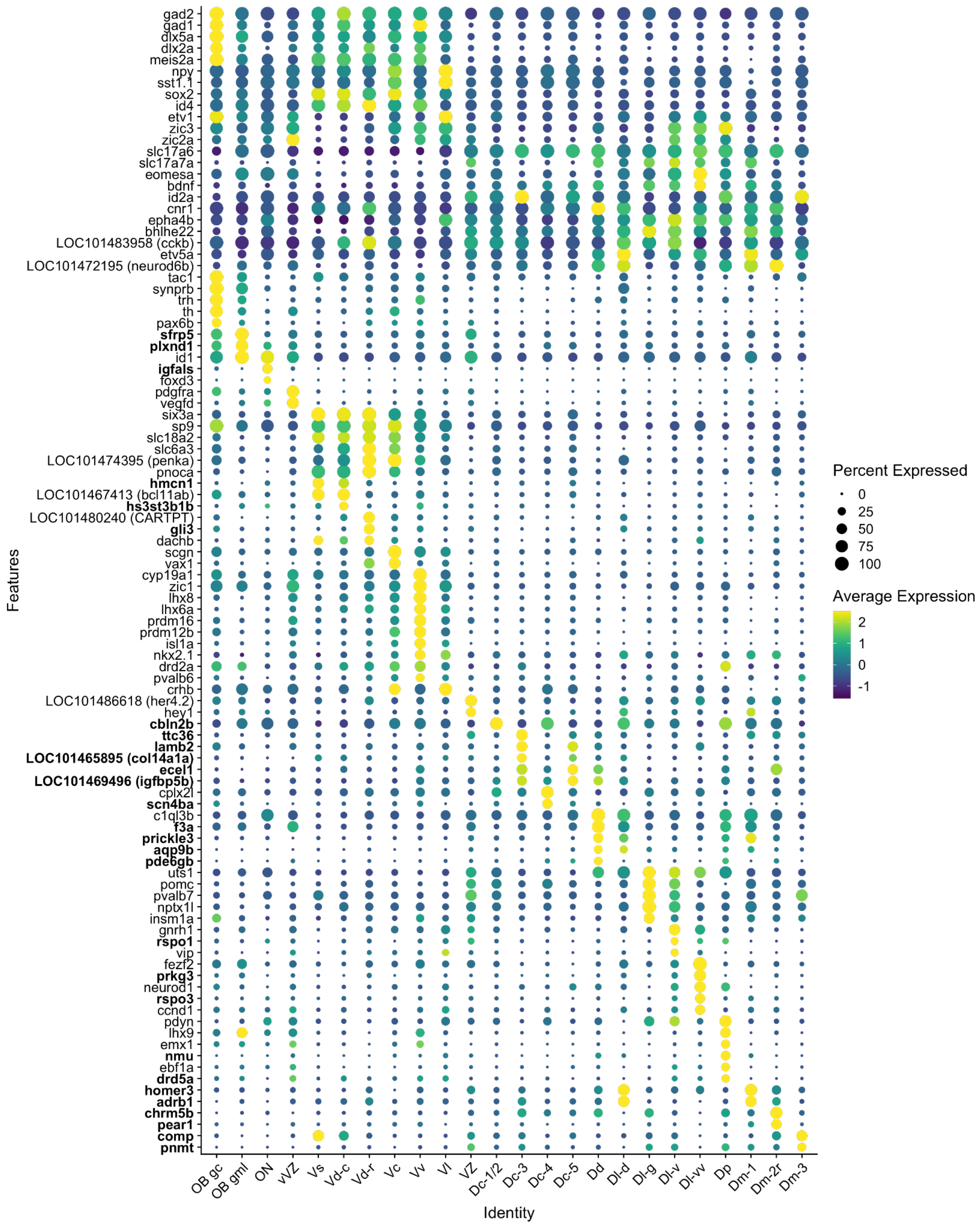

Figure S6. Gene expression of marker genes across anatomical regions in the cichlid telencephalon. Dots are colored by the average gene expression in each anatomical region and size indicates the percent of spots expressing the gene. Novel marker genes identified by

ST are bolded. Expression patterns of genes not bolded have been described previously in the teleost telencephalon.

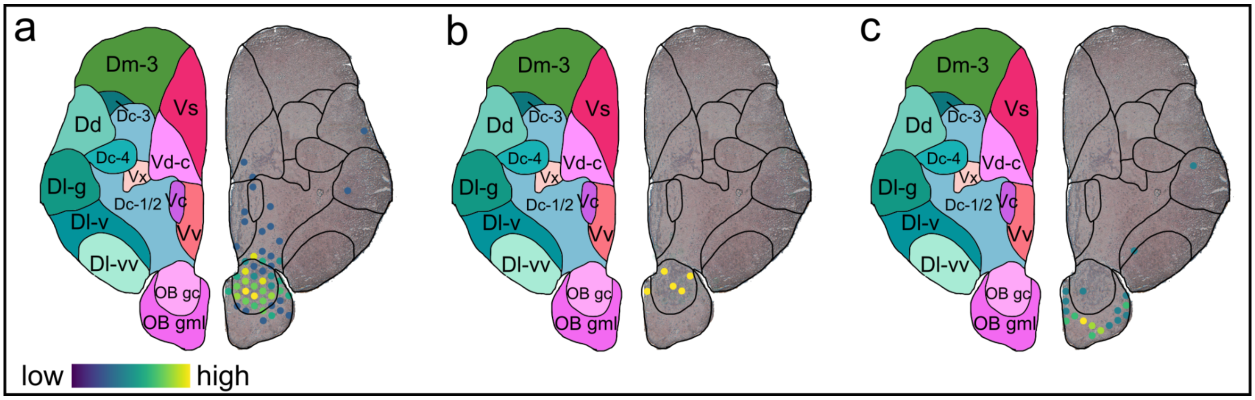

Figure S7. Gene expression in the olfactory bulb granule cell layer and glomerular/mitral cell layer. a-c) Spots are colored by the gene expression of *th*, *pax6*, and *lhx9* respectively. Spots not expressing these genes are not shown.

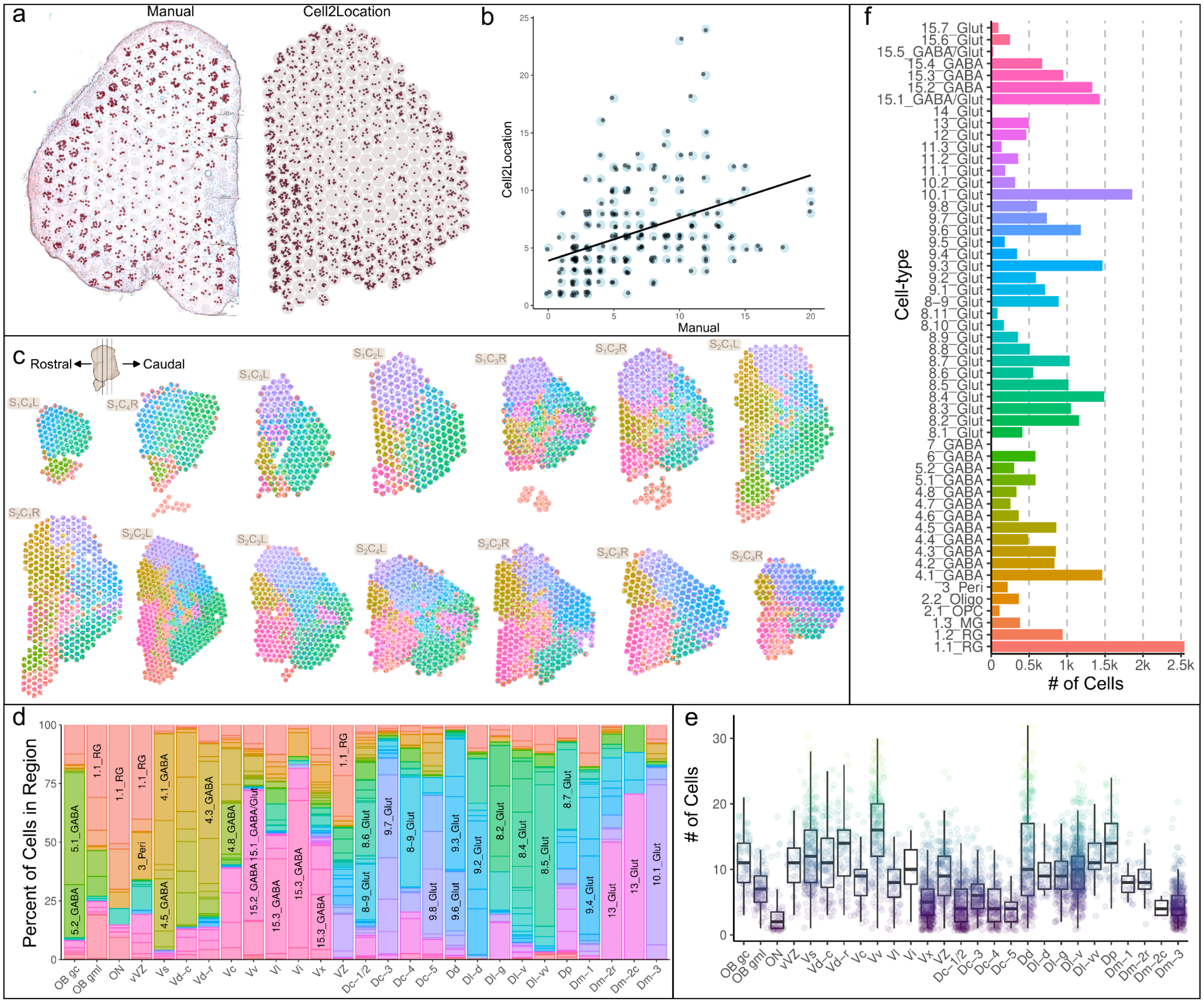

Figure S8. Cell-type location and abundance prediction. a) Comparison of manually counted estimates (left) of the number of cells per spot compared with computational estimates (right) of abundance for select spots in a select tissue hemisphere (S_1C_2R). Large circles represent spots and smaller dots within them represent estimated cells. **b)** Correlation of manual and computation estimate of number of cells for spots shown in the previous panel (Pearson's correlation coefficient=0.3989124; Pearson's correlation coefficient p-value=1.407487e-09; Spearman's correlation coefficient=0.4973912; Spearman's correlation coefficient p-value=8.943762e-15). Points are jittered slightly, within the radius of the blue circles, in order to better visualize the number of spots driving the correlation. **c)** Cell2location cell-type abundance estimates (small dots) in spots (large circles) in all tissue hemispheres. Spots are colored by the cell-type[5] with the greatest number of predicted cells within the spot (color-coding scheme is shown in panel F). **d)** Composition of anatomical regions by cell-types (color-coding scheme is shown in panel F). Cell-types comprising greater than 30% of cells in the cell-type are labeled. **e)** Number of cells in each anatomical region. **f)** Number of cells from each cell-type in all spots.

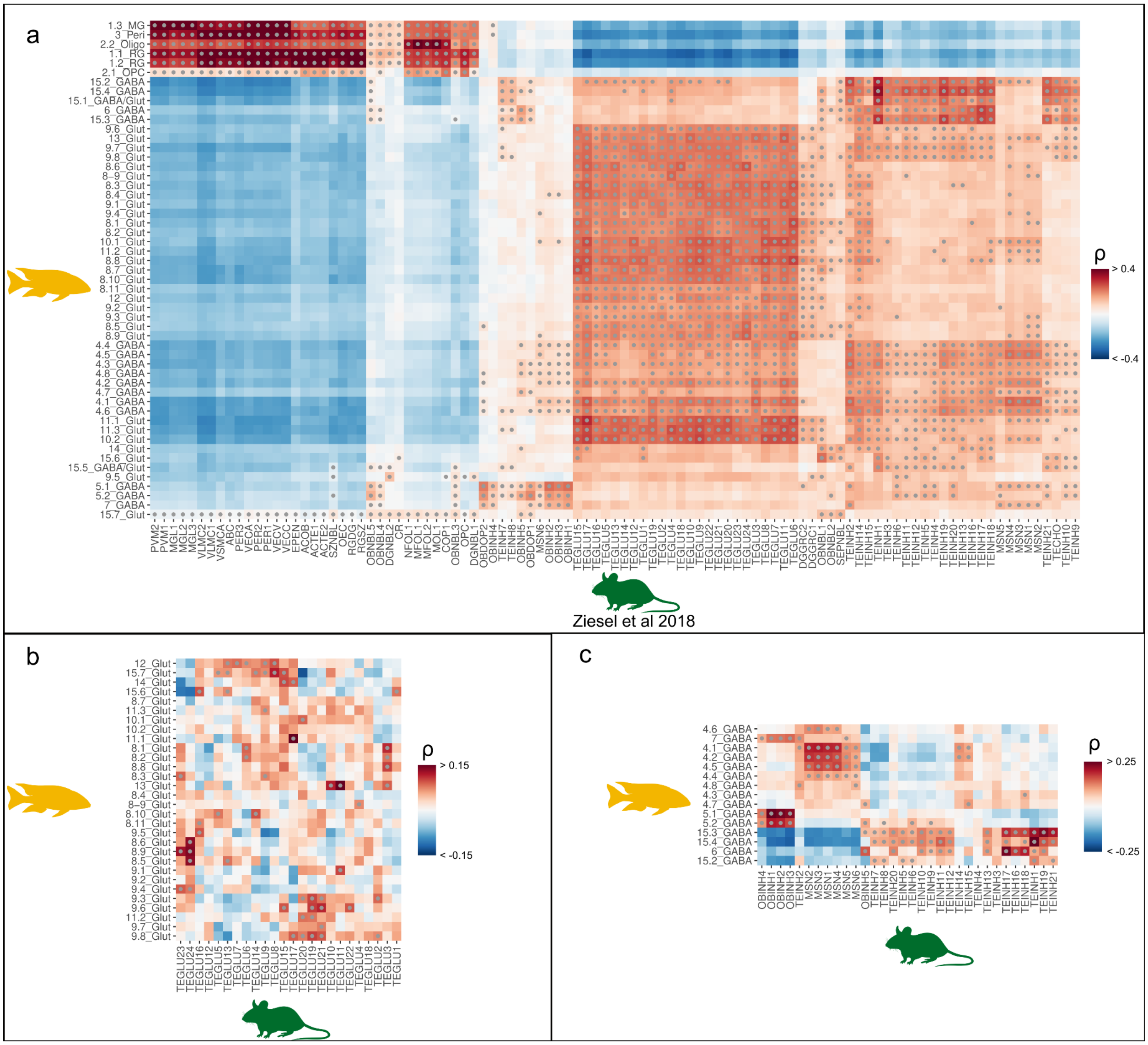

Figure S9. Correlation of gene expression profiles of shared marker genes between cichlids and mice. a) Correlation of transcriptomic profiles by cell-type of DEGs shared between cichlids[5] and mice[24]. Gray dots denote cell-type pairs with correlations significant after permutation testing (n_{perm}=10,000). **b-c)** Correlations between glutamatergic and GABAergic cichlid and mouse cell-types, respectively.

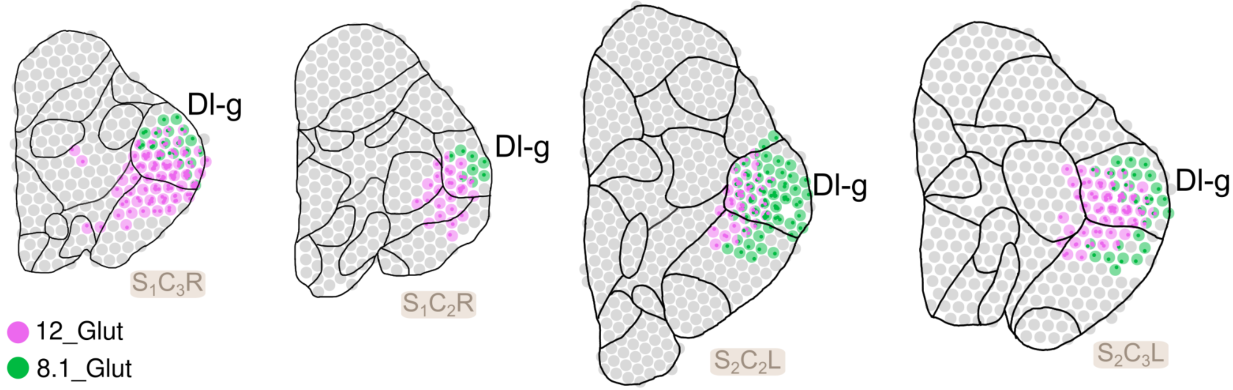

Figure S10. Predicted location of Dl-g cell-types 8.1_Glut and 12_Glut. Cell2location cell-type abundance estimates (small dots) in spots (large circles) of Dl-g in select tissue hemispheres (S_1C_3R, S_1C_2R, S_2C_2L and S_2C_3L). Spots predicted to contain 12_Glut cells are colored pink and spots predicted to contain 8.1_Glut cells are colored green. Spots predicted to both of these cell-types are colored half pink and half green.

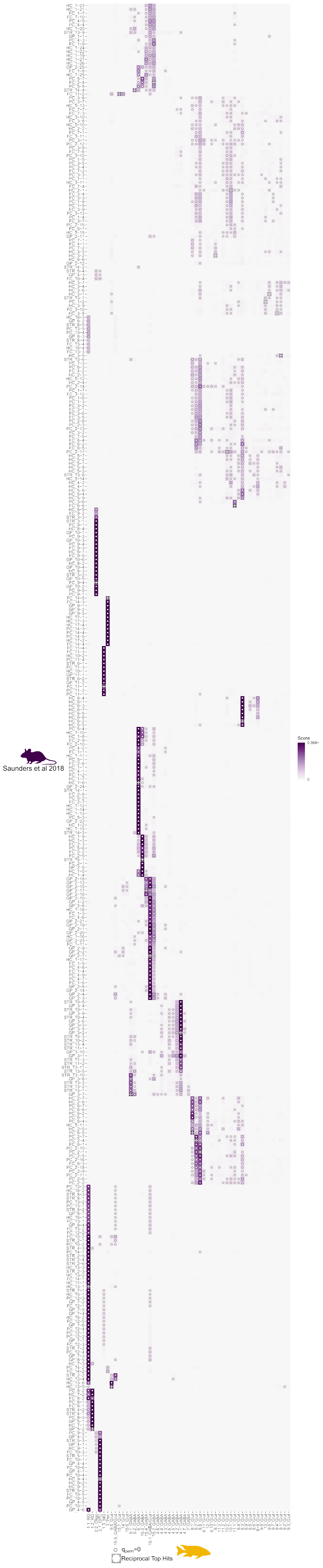

420

Figure S11. Comparison of cichlid and mouse cell-types using SAMap. Similarity score of cell-types in the cichlid[5] and mouse telencephalon (Saunders et al. 2018)[22] with dots denoting similarity scores greater than all permutations.

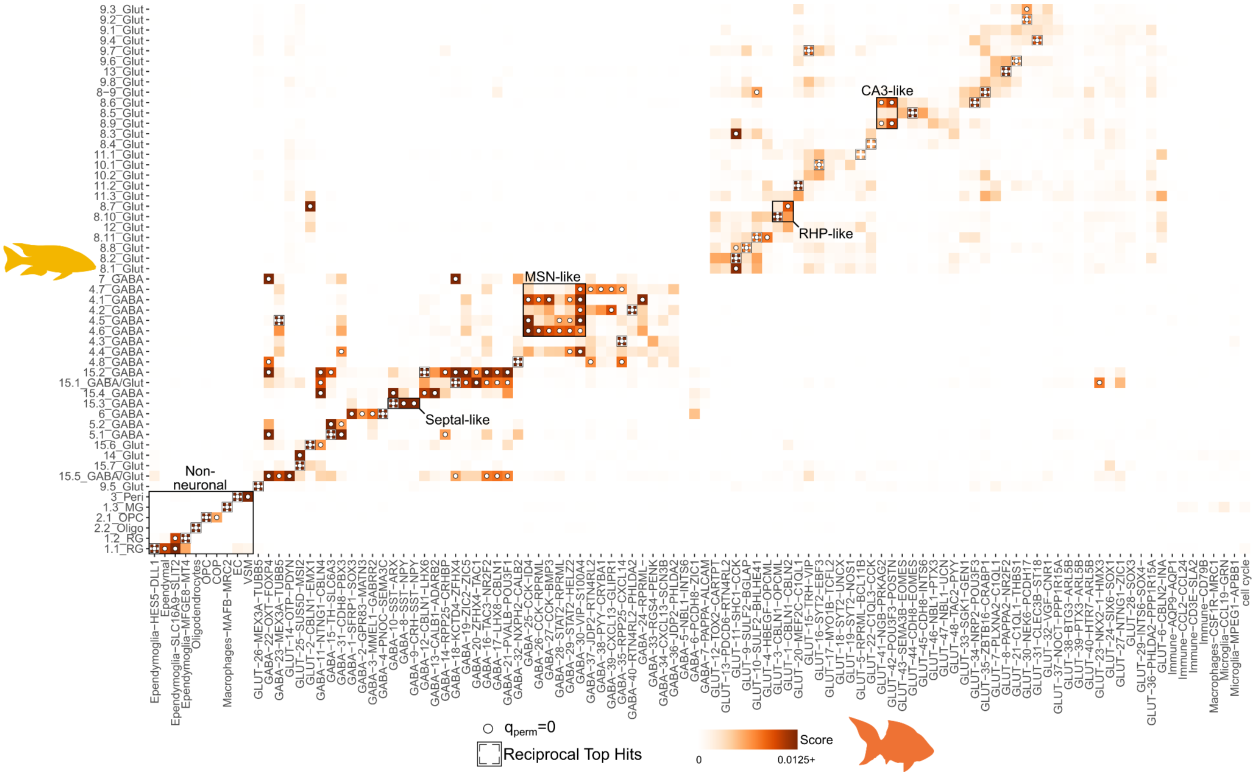

Figure S12. Comparison of cichlid and goldfish cell-types using SAMap. Similarity score of cell-types in the cichlid[5] and goldfish telencephalon[23] with dots denoting similarity scores greater than all permutations.

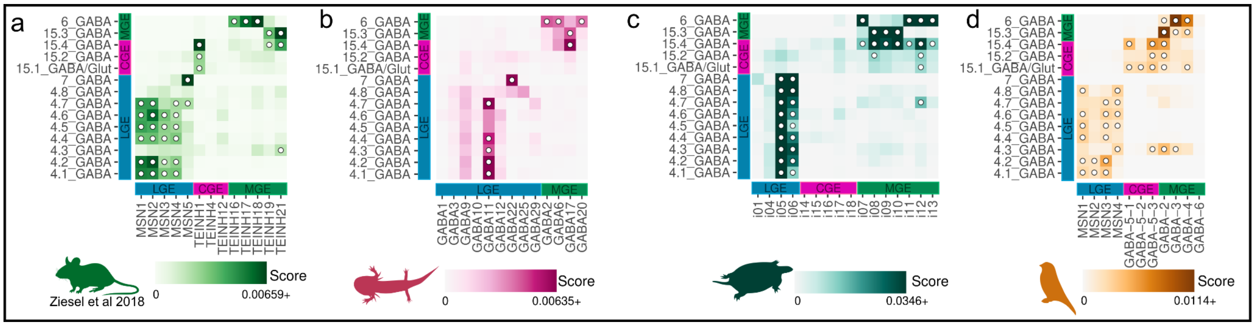

Figure S13. Comparison of GABAergic cell-types across vertebrates. a-d) Similarity scores of GABAergic cell-types in SAMap comparisons of cichlids[5] to mice[24], axolotls[45], turtles[46], and birds[47] respectively. Dots denote similarity scores greater than all permutations. Colored labels on the x-axis indicate the previously described origin of the cell-type (LGE, CGE and MGE). Colored labels on the y-axis indicate the putative origin of the cichlid cell-type.

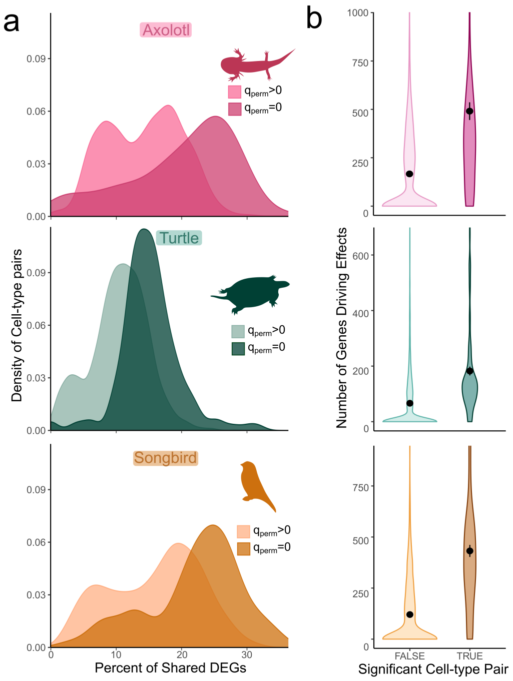

Figure S14. Genes underlying cell-type relationships in axolotls, turtles, and songbirds to cichlid fish. a) Distribution of the percent of shared DEGs between significant (q_{perm}=0) and non-significant (q_{perm}>0) cell-type pairs. Cell-types pairs with significant similarities between species have more DEGs in common than other cell-types pairs (Welch Two Sample t-test; axolotl p=2.17e-10, t=7.02; turtle p=5.54e-25, t=12; songbird p=1.63e-19, t=10.2). **b)** Violin plot of the number of genes driving effects for cell-type pairs that were significant versus non-significant. Dots indicate the mean value and vertical lines represent the standard error. Possible outliers (<1st or >99th percentile) are not visualized. Cell-type pairs with significant similarities also had significantly more SAMap driving genes than other cell-type pairs (Welch Two Sample; axolotl: p=1.79e-10, t=7.08; turtle: p=1.69e-11, t=7.27; songbird: p=5.68e-20, t=10.5).

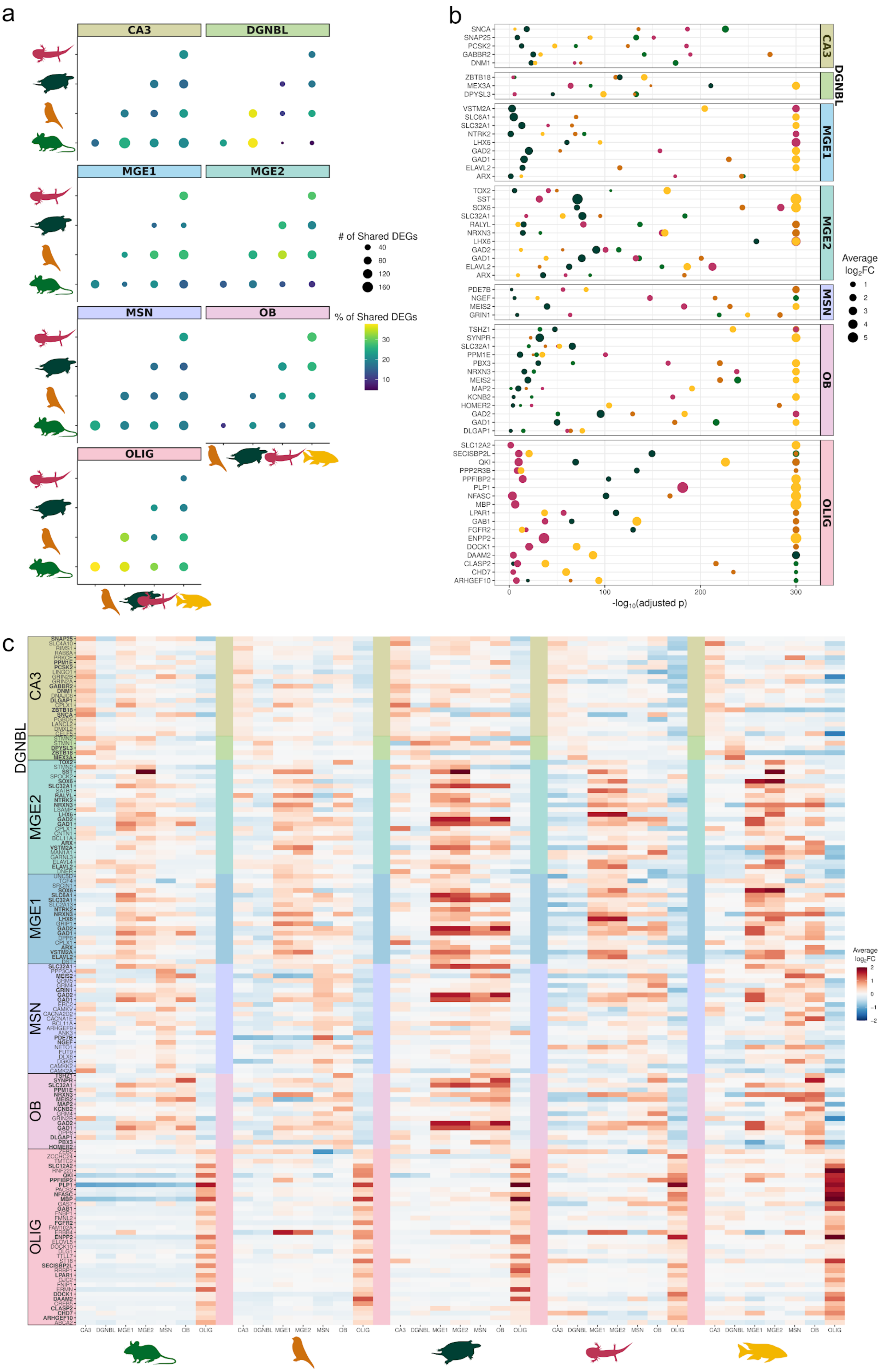
a
CA3
DGNBL
MGE1
MGE2
MSN
OB
OLIG
# of Shared DEGs
40
80
120
160
% of Shared DEGs
30
20
10
b
SNCA
SNAP25
PCSK2
GABBR2
DNM1
ZBTB18
MEX3A
DPYSL3
VSTM2A
SLC6A1
SLC32A1
NTRK2
LHX6
GAD2
GAD1
ELAVL2
ARX
TOX2
SST
SOX6
SLC32A1
RALYL
NRXN3
LHX6
GAD2
GAD1
ELAVL2
ARX
PDE7B
NGEF
MEIS2
GRIN1
TSHZ1
SYNPR
SLC32A1
PPM1E
PBX3
NRXN3
MEIS2
MAP2
KCNB2
HOMER2
GAD2
GAD1
DLGAP1
SLC12A2
SECISBP2L
QKI
PPP2R3B
PPFIBP2
PLP1
NFASC
MBP
LPAR1
GAB1
FGFR2
ENPP2
DOCK1
DAAM2
CLASP2
CHD7
ARHGEF10
0
100
200
300
-log10(adjusted p)
Average log2FC
1
2
3
4
5
c
CA3
DGNBL
MGE2
MGE1
MSN
OB
OLIG
Average log2FC
2
1
0
−1
−2
CA3 DGNBL MGE1 MGE2 MSN OB OLIG

Figure S15. Genes shared across conserved cell-types in vertebrates. a) Number and percent of shared marker genes across pairwise combinations of vertebrates in strongly conserved cell-types in all vertebrates[5,24,45–47]. Dot color signifies the percent of markers shared and dot size reflects the number of marker genes shared. **b)** Marker genes shared in all vertebrates in strongly conserved cell-types. Dot size signifies the average log_2FC of the marker and the $-log_{10}$ of the adjusted p-value is shown on the x-axis. Dot color reflects the species of origin (green=mouse, brown=bird, teal=turtle, pink=axolotl and yellow=cichlid). **c)** Heatmap of average log_2FC of marker genes (in bold text) and genes with positive log_2FC in all vertebrates. Heatmaps are shown for each species separately using the same orthologous genes.

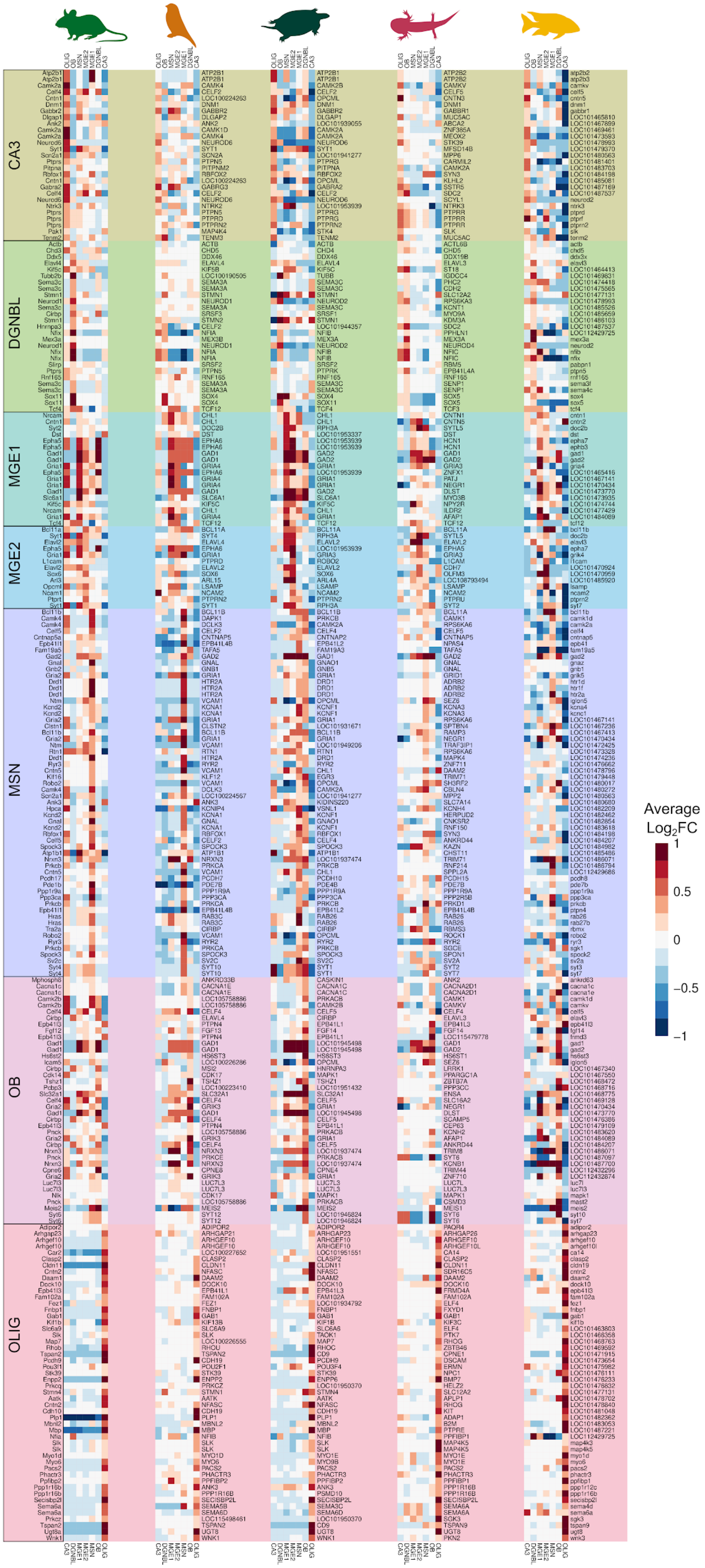
CA3
DGNBL
MGE1
MGE2
MSN
OB
OLIG
Average Log2FC
1
0.5
0
−0.5
−1

Figure S16. Genes driving conserved cell-type relationships across vertebrates. Heatmap of average log_2FC of genes driving conserved cell-types in vertebrates[5,24,45–47]. Heatmaps are shown for each species separately using genes orthologous to the same cichlid gene.

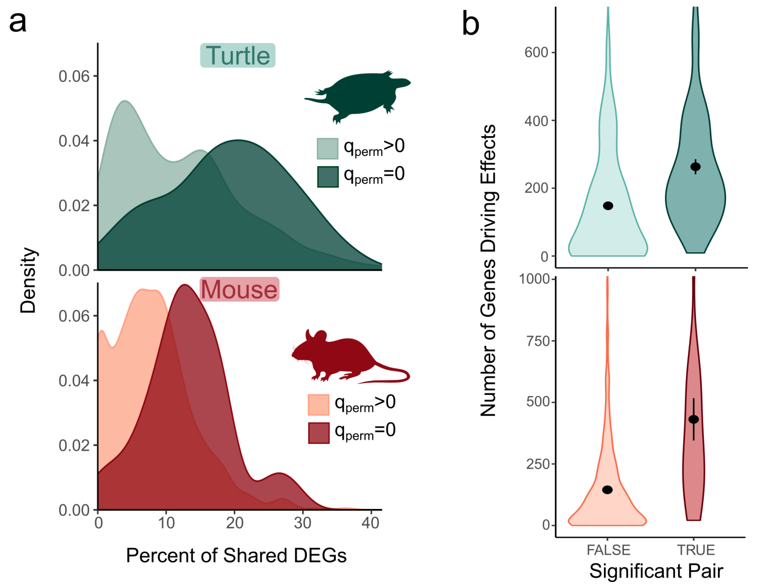

Figure S17. Genes underlying anatomical region relationships in cichlid fish and mice. a) Distribution of the percent of shared DEGs between significant (q_{perm}=0) and non-significant (q_{perm}>0) anatomical regions. Regions with significant similarities have more DEGs in common than other regions (turtle p=2.39e-07, t=5.68; mouse p=4.94e-04, t=4.03). **b)** Violin plot of the number of genes driving effects for significant versus non-significant region pairs. Dots indicate the mean value and vertical lines represent the standard error. Possible outliers (<1st or >99th percentile) are not visualized. Significant region pairs had significantly more genes driving effects than non-significant region pairs (Welch Two Sample; turtle: p=4.87e-06, t=4.89; mouse: p=0.00375, t=3.32).

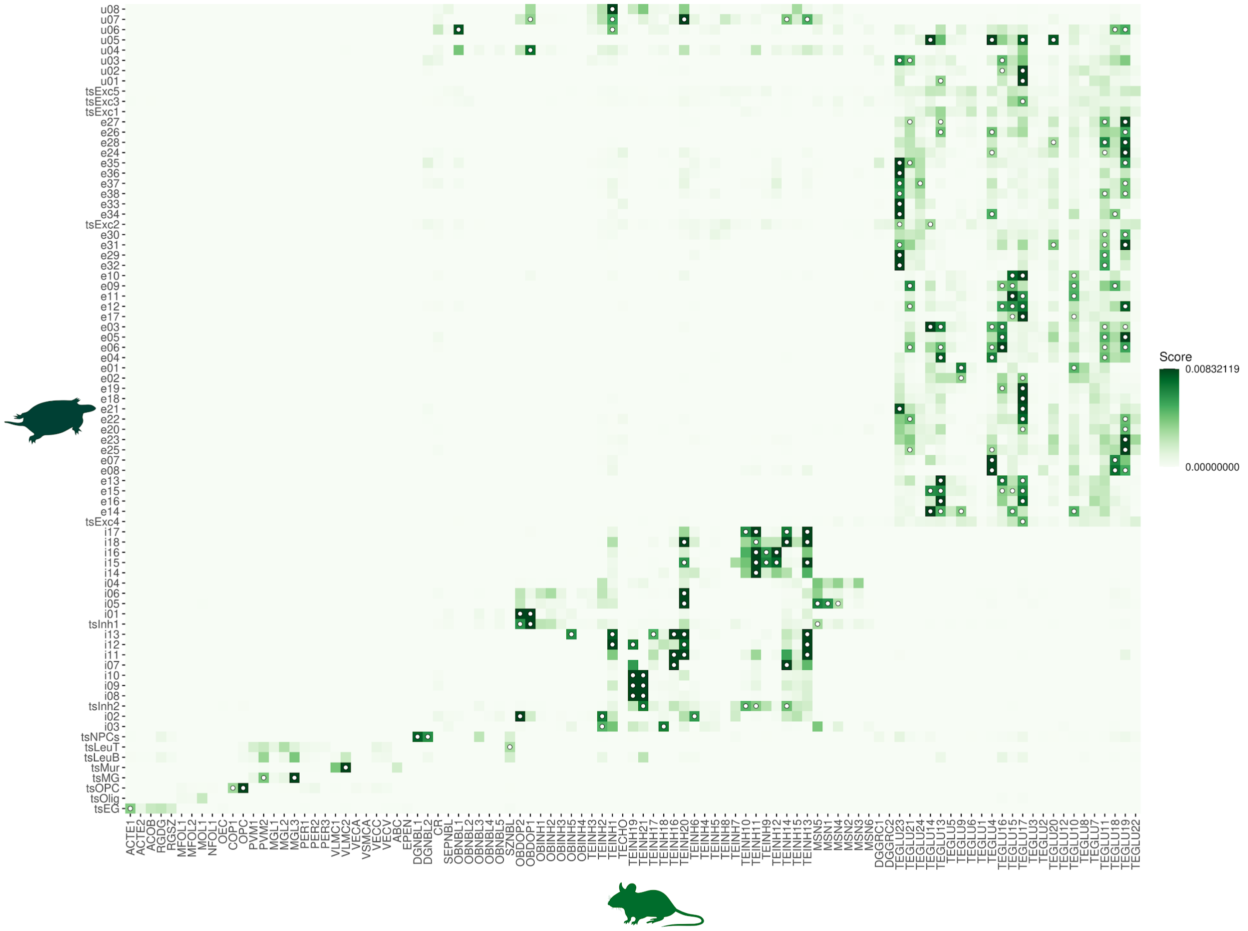

Figure S18. Comparison of turtle and mouse cell-types using SAMap. Similarity score of cell-types in the turtle[46] and mouse[24] telencephalon with dots denoting similarity scores greater than all permutations.

SUPPLEMENTARY REFERENCES

1. Burmeister, S. S., Munshi, R. G. & Fernald, R. D. Cytoarchitecture of a cichlid fish telencephalon. *Brain Behav. Evol.* **74**, 110–120 (2009).
2. Munchrath, L. A. & Hofmann, H. A. Distribution of sex steroid hormone receptors in the brain of an African cichlid fish, Astatotilapia burtoni. *J. Comp. Neurol.* **518**, 3302–3326 (2010).
3. Maruska, K. P., Butler, J. M., Field, K. E. & Porter, D. T. Localization of glutamatergic, GABAergic, and cholinergic neurons in the brain of the African cichlid fish, Astatotilapia burtoni. *J. Comp. Neurol.* **525**, 610–638 (2017).
4. Ganz, J. *et al.* Subdivisions of the adult zebrafish subpallium by molecular marker analysis. *J. Comp. Neurol.* **520**, 633–655 (2012).
5. Johnson, Z. V. *et al.* Cellular profiling of a recently-evolved social behavior in cichlid fishes. *Nat. Commun.* **14**, 1–19 (2023).
6. Diotel, N. *et al.* Comprehensive expression map of transcription regulators in the adult zebrafish telencephalon reveals distinct neurogenic niches. *J. Comp. Neurol.* **523**, 1202–1221 (2015).
7. Aoki, T. *et al.* Imaging of neural ensemble for the retrieval of a learned behavioral program. *Neuron* **78**, 881–894 (2013).
8. Website. Grone, B. P., Butler, J. M., Wayne, C. R. & Maruska, K. P. Expression patterns and evolution of urocortin and corticotropin-releasing hormone genes in a cichlid fish. Journal of Comparative Neurology vol. 529 2596–2619 Preprint at https://doi.org/10.1002/cne.25113.
9. Hu, C. K. *et al.* Identification of prohormones and pituitary neuropeptides in the African cichlid, Astatotilapia burtoni. *BMC Genomics* **17**, 660 (2016).
10. Mueller, T., Dong, Z., Berberoglu, M. A. & Guo, S. The dorsal pallium in zebrafish, Danio rerio (Cyprinidae, Teleostei). *Brain Res.* **1381**, 95–105 (2011).

11. Furlan, G. *et al.* Life-Long Neurogenic Activity of Individual Neural Stem Cells and Continuous Growth Establish an Outside-In Architecture in the Teleost Pallium. *Curr. Biol.* **27**, 3288–3301.e3 (2017).
12. Huffman, L. S. *et al.* Distribution of nonapeptide systems in the forebrain of an African cichlid fish, Astatotilapia burtoni. *J. Chem. Neuroanat.* **44**, 86–97 (2012).
13. Ogawa, S. *et al.* Cloning and expression of tachykinins and their association with kisspeptins in the brains of zebrafish. *J. Comp. Neurol.* **520**, 2991–3012 (2012).
14. Porter, D. T., Roberts, D. A. & Maruska, K. P. Distribution and female reproductive state differences in orexigenic and anorexigenic neurons in the brain of the mouth brooding African cichlid fish, Astatotilapia burtoni. *J. Comp. Neurol.* **525**, 3126–3157 (2017).
15. Pickavance, L. C., Staines, W. A. & Fryer, J. N. Distributions and colocalization of neuropeptide Y and somatostatin in the goldfish brain. *J. Chem. Neuroanat.* **5**, 221–233 (1992).
16. Yamamoto, K., Ruuskanen, J. O., Wullimann, M. F. & Vernier, P. Differential expression of dopaminergic cell markers in the adult zebrafish forebrain. *J. Comp. Neurol.* **519**, 576–598 (2011).
17. O'Connell, L. A., Fontenot, M. R. & Hofmann, H. A. Characterization of the dopaminergic system in the brain of an African cichlid fish, Astatotilapia burtoni. *J. Comp. Neurol.* **519**, 75–92 (2011).
18. Mueller, T., Wullimann, M. F. & Guo, S. Early teleostean basal ganglia development visualized by zebrafish Dlx2a, Lhx6, Lhx7, Tbr2 (eomesa), and GAD67 gene expression. *J. Comp. Neurol.* **507**, 1245–1257 (2008).
19. Alunni, A. *et al.* Cloning and developmental expression patterns of Dlx2, Lhx7 and Lhx9 in the medaka fish (Oryzias latipes). *Mech. Dev.* **121**, 977–983 (2004).
20. Diotel, N., Beil, T., Strähle, U. & Rastegar, S. Differential expression of id genes and their potential regulator znf238 in zebrafish adult neural progenitor cells and neurons suggests

distinct functions in adult neurogenesis. *Gene Expr. Patterns* **19**, 1–13 (2015).

21. Kleshchevnikov, V. *et al.* Cell2location maps fine-grained cell types in spatial transcriptomics. *Nat. Biotechnol.* **40**, 661–671 (2022).
22. Saunders, A. *et al.* Molecular Diversity and Specializations among the Cells of the Adult Mouse Brain. *Cell* **174**, 1015–1030.e16 (2018).
23. Tibi, M. *et al.* A telencephalon cell type atlas for goldfish reveals diversity in the evolution of spatial structure and cell types. *Sci Adv* **9**, eadh7693 (2023).
24. Zeisel, A. *et al.* Molecular Architecture of the Mouse Nervous System. *Cell* **174**, 999–1014.e22 (2018).
25. Edsgärd, D., Johnsson, P. & Sandberg, R. Identification of spatial expression trends in single-cell gene expression data. *Nat. Methods* **15**, 339–342 (2018).
26. Yoshihara, S.-I., Omichi, K., Yanazawa, M., Kitamura, K. & Yoshihara, Y. Arx homeobox gene is essential for development of mouse olfactory system. *Development* **132**, 751–762 (2005).
27. Heng, X. *et al.* Sall3 correlates with the expression of TH in mouse olfactory bulb. *J. Mol. Neurosci.* **46**, 293–302 (2012).
28. Pignatelli, A. & Belluzzi, O. Neurogenesis in the Adult Olfactory Bulb. in *The Neurobiology of Olfaction* (ed. Menini, A.) (CRC Press/Taylor & Francis, Boca Raton (FL)).
29. Gokce, O. *et al.* Cellular Taxonomy of the Mouse Striatum as Revealed by Single-Cell RNA-Seq. *Cell Rep.* **16**, 1126–1137 (2016).
30. Risold, P. Y. & Swanson, L. W. Chemoarchitecture of the rat lateral septal nucleus. *Brain Res. Brain Res. Rev.* **24**, 91–113 (1997).
31. Ikeda, K. *et al.* Distribution of prepro-nociceptin/orphanin FQ mRNA and its receptor mRNA in developing and adult mouse central nervous systems. *J. Comp. Neurol.* **399**, 139–151 (1998).
32. Uchigashima, M., Cheung, A., Suh, J., Watanabe, M. & Futai, K. Differential expression of

neurexin genes in the mouse brain. *J. Comp. Neurol.* **527**, 1940–1965 (2019).
33. Mühlfriedel, S., Kirsch, F., Gruss, P., Chowdhury, K. & Stoykova, A. Novel genes differentially expressed in cortical regions during late neurogenesis. *Eur. J. Neurosci.* **26**, 33–50 (2007).
34. Developmental specification of forebrain cholinergic neurons. *Dev. Biol.* **421**, 1–7 (2017).
35. Rajendram, R., Preedy, V. R., Patel, V. B. & Martin, C. R. *The Neurobiology, Physiology, and Psychology of Pain*. (Academic Press, 2021).
36. Wei, B. *et al.* The onion skin-like organization of the septum arises from multiple embryonic origins to form multiple adult neuronal fates. *Neuroscience* **222**, 110–123 (2012).
37. Inoue, T., Ota, M., Ogawa, M., Mikoshiba, K. & Aruga, J. Zic1 and Zic3 regulate medial forebrain development through expansion of neuronal progenitors. *J. Neurosci.* **27**, 5461–5473 (2007).
38. Available from mouse.brain-map.org.Allen Institute for Brain Science (2011).
39. Zhao, Y. *et al.* The LIM-homeobox gene Lhx8 is required for the development of many cholinergic neurons in the mouse forebrain. *Proc. Natl. Acad. Sci. U. S. A.* **100**, 9005–9010 (2003).
40. Bonaventure, P. *et al.* Nuclei and subnuclei gene expression profiling in mammalian brain. *Brain Res.* **943**, 38–47 (2002).
41. Cembrowski, M. S., Wang, L., Sugino, K., Shields, B. C. & Spruston, N. Hipposeq: a comprehensive RNA-seq database of gene expression in hippocampal principal neurons. *Elife* **5**, e14997 (2016).
42. Lein, E. S. *et al.* Genome-wide atlas of gene expression in the adult mouse brain. *Nature* **445**, 168–176 (2006).
43. Belgard, T. G. *et al.* A transcriptomic atlas of mouse neocortical layers. *Neuron* **71**, 605–616 (2011).
44. Butler, A. B. & Hodos, W. *Comparative Vertebrate Neuroanatomy: Evolution and*

Adaptation. (John Wiley & Sons, 2005).

45. Lust, K. *et al.* Single-cell analyses of axolotl telencephalon organization, neurogenesis, and regeneration. *Science* **377**, eabp9262 (2022).
46. Tosches, M. A. *et al.* Evolution of pallium, hippocampus, and cortical cell types revealed by single-cell transcriptomics in reptiles. *Science* **360**, 881–888 (2018).
47. Colquitt, B. M., Merullo, D. P., Konopka, G., Roberts, T. F. & Brainard, M. S. Cellular transcriptomics reveals evolutionary identities of songbird vocal circuits. *Science* **371**, (2021).
