# Supplementary material for: Spatially resolved cell atlas of the teleost telencephalon and deep homology of the vertebrate forebrain
Source: Commun Biol. 2024 May 21;7:612. doi: 10.1038/s42003-024-06315-1 (PMC11109250; doi:10.1038/s42003-024-06315-1)
Supplement: Supplementary file 3 — Description of Additional Supplementary Files [file 42003_2024_6315_MOESM3_ESM.pdf]

Description of Additional Supplementary Files

File name: Supplementary Data 1
Description: Summary data for subjects (Sheet 1) and sequencing information for each capture area (Sheet 2).

File name: Supplementary Data 2
Description: Silhouette scores of bootstraps of the data from ChooseR, including the 95% confidence interval and median of the silhouette scores. These scores are shown for all combinations of parameters tested and include the number of resulting clusters.

File name: Supplementary Data 3
Description: Summary data of unbiased clusters, including the highly variable genes that formed the clusters (Sheet 1), the number of spots from each cluster in each tissue hemisphere (Sheet 2), the top 5 markers of clusters (Sheet 3) and all cluster markers (Sheet 4).

File name: Supplementary Data 4
Description: Manual anatomical annotations of each spot (Sheet 1) and differentially expressed genes in the manually annotated anatomical regions (Sheet 2).

File name: Supplementary Data 5
Description: Cell-type abundance estimates by cell2location in each spot.

File name: Supplementary Data 6
Description: Results from SAMap comparative analysis of cell-types in the cichlid and mouse telencephalon. The similarity score and p-value from permutations of the data are provided.

File name: Supplementary Data 7
Description: Conserved marker genes (Sheet 1) and genes driving effects (Sheet 2) in significant cell-pairs in the cichlid and mouse telencephalon.

File name: Supplementary Data 8
Description: Results from SAMap comparative analysis of cell-types in the axolotl (Sheet 1), turtle (Sheet 2) and songbird (Sheet 3) forebrain with the cichlid telencephalon.

File name: Supplementary Data 9
Description: Conserved marker genes in significant cell-pairs in the axolotl (Sheet 1), turtle (Sheet 2) and songbird (Sheet 3) forebrain with the cichlid telencephalon. Genes driving effects in significant cell-pairs in the axolotl (Sheet 4), turtle (Sheet 5) and songbird (Sheet 6) forebrain with the cichlid telencephalon.

File name: Supplementary Data 10
Description: Conserved marker genes and genes with positive logFC values without requiring differential expression (Sheet 1) and genes driving effects (Sheet 2) in strongly conserved cell-types in all vertebrates.

File name: Supplementary Data 11

Description: Results from SAMap comparative analysis of brain regions in the turtle forebrain (Sheet 1) and mouse telencephalon (Sheet 2) with the cichlid telencephalon

File name: Supplementary Data 12

Description: Conserved marker genes in significant brain region pairs in the turtle forebrain (Sheet 1) and mouse telencephalon (Sheet 2) with the cichlid telencephalon. Genes driving effects in significant brain region pairs in the turtle forebrain (Sheet 1) and mouse telencephalon (Sheet 2).

File name: Supplementary Data 13

Description: Composition of genes driving significant cell-type pairs in neocortical-like structures by gene categories in the turtle, cichlid and mouse forebrain (Sheet 1). Gene categories for which the composition was examined (Sheet 2). These categories include transcription factors, neuromodulatory ligands and receptors.

File name: Supplementary Data 14

Description: Source data for all graphs presented in the main figures.

File name: Supplementary Data 14

Description:
