# Supplementary material for: Spatially resolved cell atlas of the teleost telencephalon and deep homology of the vertebrate forebrain
Source: Commun Biol. 2024 May 21;7:612. doi: 10.1038/s42003-024-06315-1 (PMC11109250; doi:10.1038/s42003-024-06315-1)
Supplement: Supplementary file 18 — Reporting Summary [file 42003_2024_6315_MOESM18_ESM.pdf]

nature portfolio

Corresponding author(s): George Gruenhagen
Jeffrey Todd Streelman

Last updated by author(s): Apr 29, 2024

# Reporting Summary

Nature Portfolio wishes to improve the reproducibility of the work that we publish. This form provides structure for consistency and transparency in reporting. For further information on Nature Portfolio policies, see our Editorial Policies and the Editorial Policy Checklist.

## Statistics

For all statistical analyses, confirm that the following items are present in the figure legend, table legend, main text, or Methods section.

| n/a | Confirmed | |
|---|---|---|
| ☐ | ☒ | The exact sample size (*n*) for each experimental group/condition, given as a discrete number and unit of measurement |
| ☐ | ☒ | A statement on whether measurements were taken from distinct samples or whether the same sample was measured repeatedly |
| ☐ | ☒ | The statistical test(s) used AND whether they are one- or two-sided<br>*Only common tests should be described solely by name; describe more complex techniques in the Methods section.* |
| ☐ | ☒ | A description of all covariates tested |
| ☐ | ☒ | A description of any assumptions or corrections, such as tests of normality and adjustment for multiple comparisons |
| ☐ | ☒ | A full description of the statistical parameters including central tendency (e.g. means) or other basic estimates (e.g. regression coefficient) AND variation (e.g. standard deviation) or associated estimates of uncertainty (e.g. confidence intervals) |
| ☐ | ☒ | For null hypothesis testing, the test statistic (e.g. $F$, $t$, $r$) with confidence intervals, effect sizes, degrees of freedom and $P$ value noted<br>*Give P values as exact values whenever suitable.* |
| ☐ | ☒ | For Bayesian analysis, information on the choice of priors and Markov chain Monte Carlo settings |
| ☐ | ☒ | For hierarchical and complex designs, identification of the appropriate level for tests and full reporting of outcomes |
| ☐ | ☒ | Estimates of effect sizes (e.g. Cohen's *d*, Pearson's *r*), indicating how they were calculated |

*Our web collection on statistics for biologists contains articles on many of the points above.*

## Software and code

Policy information about availability of computer code

| | |
|---|---|
| Data collection | No software was used. |
| Data analysis | Custom code for data analysis is here https://github.com/ggruenhagen3/cichlid_st and a Zenodo doi for the version of the code at the time of submission is 10.5281/zenodo.8364925. |

For manuscripts utilizing custom algorithms or software that are central to the research but not yet described in published literature, software must be made available to editors and reviewers. We strongly encourage code deposition in a community repository (e.g. GitHub). See the Nature Portfolio guidelines for submitting code & software for further information.

## Data

Policy information about availability of data

All manuscripts must include a data availability statement. This statement should provide the following information, where applicable:

- Accession codes, unique identifiers, or web links for publicly available datasets
- A description of any restrictions on data availability
- For clinical datasets or third party data, please ensure that the statement adheres to our policy

All data needed to evaluate the conclusions in this paper are present in the paper and/or the Supplementary Materials. Custom scripts for data analyses can be found at GitHub (https://github.com/ggruenhagen3/cichlid_st) and Zenodo (10.5281/zenodo.8364925).

## Human research participants

Policy information about studies involving human research participants and Sex and Gender in Research.

| | |
|---|---|
| Reporting on sex and gender | N/A |
| Population characteristics | N/A |
| Recruitment | N/A |
| Ethics oversight | N/A |

Note that full information on the approval of the study protocol must also be provided in the manuscript.

# Field-specific reporting

Please select the one below that is the best fit for your research. If you are not sure, read the appropriate sections before making your selection.

☒ Life sciences ☐ Behavioural & social sciences ☐ Ecological, evolutionary & environmental sciences

For a reference copy of the document with all sections, see nature.com/documents/nr-reporting-summary-flat.pdf

# Life sciences study design

All studies must disclose on these points even when the disclosure is negative.

| | |
|---|---|
| Sample size | Thousands of spatial spots captured RNA transcripts from the brains of multiple fishes (n=2). Comparisons of transcriptomics datasets were conducted with thousands of spatial spots, single cells, or single nuclei. |
| Data exclusions | High quality transcriptomic data from the forebrain was used in this study. Generally, quality metrics for the transcriptomics data evaluated how much RNA was captured, more details can be found in the Methods. |
| Replication | Findings described in this study are based on analysis of transcriptomics data from multiple fishes. Additionally, when possible, findings were corroborated by analysis using multiple methods and datasets. |
| Randomization | There were not experimental groups in this study. |
| Blinding | There were not experimental groups in this study. |

# Reporting for specific materials, systems and methods

We require information from authors about some types of materials, experimental systems and methods used in many studies. Here, indicate whether each material, system or method listed is relevant to your study. If you are not sure if a list item applies to your research, read the appropriate section before selecting a response.

## Materials & experimental systems

| n/a | Involved in the study | |
|---|---|---|
| ☒ | ☐ | Antibodies |
| ☒ | ☐ | Eukaryotic cell lines |
| ☒ | ☐ | Palaeontology and archaeology |
| ☐ | ☒ | Animals and other organisms |
| ☒ | ☐ | Clinical data |
| ☒ | ☐ | Dual use research of concern |

## Methods

| n/a | Involved in the study | |
|---|---|---|
| ☒ | ☐ | ChIP-seq |
| ☒ | ☐ | Flow cytometry |
| ☒ | ☐ | MRI-based neuroimaging |

## Animals and other research organisms

Policy information about studies involving animals; ARRIVE guidelines recommended for reporting animal research, and Sex and Gender in Research

| | |
|---|---|
| Laboratory animals | Adult Mchenga conophoros cichlids were used in this study. |

| | |
|---|---|
| Wild animals | This study did not involve wild animals. |
| Reporting on sex | Male Mchenga conophoros cichlids were used in this study. Sex was determined by visual inspection. |
| Field-collected samples | This study did not involve samples collected from the field. |
| Ethics oversight | This study was conducted in accordance with the Institutional Animal Care and Use Committee guidelines (IACUC protocol number A100029). |

Note that full information on the approval of the study protocol must also be provided in the manuscript.
